# Primary Health Care Coverage and Child Mortality in Sub-Saharan Africa

**DOI:** 10.1001/jamanetworkopen.2026.27655

**Published:** 2026-08-07

**Authors:** Gonzalo Barreix, Sophie ter Braak, Daiana Albino Pena, Andrea Ferreira da Silva, Elisa Landin, Lucas Sales, Rodrigo Anderle, Ivalda Macicame, James Macinko, Renato Tasca, Inácio Mandomando, Sintayehu Wolka, Selidji Todagbe Agnandji, Nega Assefa, Bryony Simmons, Bàrbara Baro, Quique Bassat, Davide Rasella

**Affiliations:** 1 Barcelona Institute for Global Health (ISGlobal), Barcelona, Spain; 2 Facultat de Medicina i Ciències de la Salut, Universitat de Barcelona, Barcelona, Spain; 3 University Medical Center Utrecht, Utrecht, the Netherlands; 4 Institute of Collective Health, Federal University of Bahia, Bahia, Brazil; 5 Universidade Regional do Cariri, Cariri, Brazil; 6 Instituto Nacional de Saúde, Maputo, Mozambique; 7 University of California, Los Angeles, Los Angeles; 8 Institute for Health Policies Studies, Rio de Janeiro, Brazil; 9 Centro de Investigação em Saúde de Manhiça, Maputo, Mozambique; 10 Ministry of Health, Federal Democratic Republic of Ethiopia, Addis Ababa, Ethiopia; 11 Centre de Recherches Médicales de Lambaréné, Lambaréné, Gabon; 12 Institut für Tropenmedizin, Eberhard-Karls-Universität Tübingen, Tübingen, Germany; 13 College of Health and Medical Sciences, Haramaya University, Harar, Ethiopia; 14 LSE Health, London School of Economics & Political Science, London, United Kingdom; 15 ICREA, Barcelona, Spain; 16 Institut Clínic de Medicina I Dermatologia, Hospital Clínic de Barcelona, Barcelona, Spain; 17 Pediatrics Department, Hospital Sant Joan de Déu, Universitat de Barcelona, Esplugues, Barcelona, Spain; 18 CIBER de Epidemiología y Salud Pública, Instituto de Salud Carlos III, Madrid, Spain

## Abstract

**Importance:**

Primary health care (PHC) is recognized as pivotal for achieving universal health coverage and reducing mortality rates among children younger than 5 years.

**Objectives:**

To estimate the association of effective PHC coverage with child mortality in 3 diverse sub-Saharan countries over the past 2 decades and to estimate the number of child deaths that could occur by 2035 if PHC services are dismantled due to the current decrease in development assistance for health funding.

**Design, Setting, and Participants:**

This cohort study assessed children younger than 5 years from January 1, 2007, to December 31, 2022, in Mozambique, Gabon, and Ethiopia. The study drew on 5 national and subregional representative US Agency for International Development Demographic and Health surveys with longitudinal information from the 3 countries. Statistical analysis was performed from October 2024 to May 2026.

**Exposure:**

An effective PHC coverage index (PHC index) was developed for this study, integrating core dimensions of child PHC service provision and access and structured into progressively ordered coverage categories.

**Main Outcomes and Measures:**

The outcome was child mortality, assessed with 2-way fixed-effects multivariable Poisson regression models with child-clustered standard errors, adjusted for relevant confounding factors at the individual, regional, and national levels. This evaluation was also integrated into validated microsimulation forecasting analysis.

**Results:**

Of the 118 911 child-year observations of 37 583 children (mean [SD] age, 1.4 [1.3] years; 60 252 boys [50.7%]), Gabon had the greatest coverage of service provision, followed by Mozambique and Ethiopia, though Mozambique showed a marginally higher proportion in the highest category (Gabon, 796 of 33 269 [2.4%]); Mozambique, 554 of 20 728 [2.7%]; and Ethiopia, 317 of 64 914 [0.5%]); the inverse pattern was observed for the lowest coverage categories (Gabon, 2143 of 33 269 [6.4%]; Mozambique, 1976 of 20 728 [9.5%]; and Ethiopia, 32 189 of 64 914 [49.6%]). Across all child-year observations, there were 2166 deaths (1.8%). Ethiopia accounted for 2.1% of deaths (1338 of 64 914 child-years), Mozambique for 1.8% (364 of 20 728), and Gabon for 1.4% (464 of 33 269). Increasing PHC coverage was associated with decreases in child mortality in a dose-response manner, reaching a 58% reduction (rate ratio, 0.42 [95% CI, 0.19-0.91]) for consolidated high coverage. The dismantling of PHC coverage, triggered by the defunding of development assistance for health , may lead to an increase in preventable child deaths, amounting to an estimated 373 108 deaths (95% uncertainty interval, 313 152-444 244 deaths) across the 2025 to 2035 period.

**Conclusions and Relevance:**

This cohort study found evidence indicating that PHC was associated with substantially reduced mortality rates among children younger than 5 years in sub-Saharan Africa over the past 2 decades. These findings suggest that PHC should be expanded rather than dismantled, particularly amid the ongoing defunding of development assistance for health.

## Key Points

**Question:**

What has been the association between primary health care (PHC) coverage and child mortality over the past 2 decades in Mozambique, Gabon, and Ethiopia?

**Findings:**

In this cohort study including 37 583 children from 3 diverse sub-Saharan countries, PHC coverage was associated with lower rates of child mortality, following a clear dose-response gradient, reaching a 58% reduction in mortality with consolidated high PHC coverage. Projected PHC dismantling may result in 373 108 preventable child deaths by 2035.

**Meaning:**

This study suggests that expanding PHC coverage in sub-Saharan countries may be associated with a reduction in child mortality, while its dismantling could be associated with an increase in preventable child deaths.

## Teaser

This cohort study uses US Agency for International Development data to estimate the association of effective primary health care coverage with child mortality in Mozambique, Gabon, and Ethiopia over the past 2 decades and to estimate the number of child deaths that could occur by 2035 if primary health care services are dismantled.

## Introduction

The global number of child deaths among children younger than 5 years significantly decreased from 13.0 million in 1990 to 4.8 million in 2023.^1^ Despite this, the proportion of these deaths occurring in sub-Saharan Africa (SSA) increased sharply—from 29.5% in 1990 to 56.2% in 2023.^1^ The Sustainable Development Goal 3.2 targets a reduction in the mortality rate among children younger than 5 years to no more than 25 deaths per 1000 live births by 2030.^2^ As of 2023, the estimated mortality rate among children younger than 5 years in SSA remained above this target, at 69 deaths per 1000 live births, while the global estimate is 37 deaths per 1000 live births.^1^

The role of primary health care (PHC) in achieving universal health coverage was first widely recognized with the Alma-Ata Declaration in 1978 and consistently reinforced across the past decades until the Astana Declaration in 2018, emphasizing the need to progress toward its aspirational goal.^2,3^ The World Health Organization (WHO) states that PHC can address up to 90% of universal health coverage.^4^

SSA is likely the region with the most underresourced PHC systems globally, characterized by the limited availability of comprehensive health service packages for the population.^5^ Current decreases in international development assistance for health (DAH) risk further weakening these systems, with emerging evidence already showing a measurable deterioration in the delivery of PHC services.^6,7,8,9,10^

Existing literature already offers some indication about the importance of PHC in reducing child mortality.^11,12,13^ A recent study in Latin America shows that PHC nominal coverage could significantly contribute to the reduction in the mortality rate among children younger than 5 years by providing accessible, fundamental medical services that facilitate early intervention and preventive care, particularly in maternal, neonatal, and child health.^11^ In addition, there are sparse studies on the effectiveness of specific maternal and child health interventions in reducing mortality among different subgroups of children younger than 5 years.^14,15,16,17,18,19^ However, there is limited evidence on how PHC coverage has contributed to decreases in child mortality in SSA over recent decades (eAppendix 1.3 in Supplement 1).

This study estimates the association between effective PHC coverage and child mortality over almost 2 decades in 3 diverse SSA countries: Mozambique, Ethiopia, and Gabon (eAppendix 1.2 in Supplement 1). In addition, we conducted a forecasting analysis to assess how dismantling PHC services, triggered by current DAH defunding scenarios, could affect child mortality through 2035.

## Methods

This cohort study used anonymized, publicly accessible secondary data provided by the Demographic and Health Surveys (DHS) Program of the US Agency for International Development (USAID). Ethical approval for DHS data collection is granted by the ICF institutional review board and by national ethics committees in each survey country. As the data are fully deidentified and publicly released, this study did not require further ethical review. All respondents provided written informed consent to DHS at the time of the original survey. This study followed the Strengthening the Reporting of Observational Studies in Epidemiology (STROBE) reporting guideline.

### Study Design

The first part of the study consisted of an observational longitudinal design, based on data from 37 583 children younger than 5 years, from January 1, 2007, to December 31, 2022. Nationally representative child-level data from Mozambique, Gabon, and Ethiopia were obtained through the DHS program of USAID.^20^ The data were restructured into a longitudinal dataset. The follow-up period for each individual started with the birth of the child and stopped with the death of the child, when the child was 5 years of age, or when the survey year had been reached (eAppendix 2.1 in Supplement 1). Aggregated data were integrated into the individual-level information, resulting in a multilevel data structure with a total of 118 911 person-years.

### Outcome

The outcome in the analysis was child mortality. For each year, survival was assessed by determining whether the child was alive or had died, based on the recorded month and year of death, when applicable.

### Exposure

PHC coverage was assessed using an index (PHC index) composed of essential PHC services and an access dimension variable.^21^ The components of the PHC index are acknowledged as essential elements of the WHO PHC monitoring framework^22^ and are also aligned with the WHO Reproductive, Maternal, Newborn, and Child Health Universal Health Coverage component indicator^5^ and with the Sustainable Development Goal health-related indicators.^23^ The PHC index quantified the services each child effectively received, rather than using a nominal and/or aggregated-level indicator based on infrastructure or workforce availability, as in previous studies.^11,12^ The services included in the PHC index pertain to fundamental health care services for children younger than 5 years structurally tied to integrated PHC systems: (1) perceived barriers, including distance and financial constraints, representing health care accessibility; (2) family planning, signifying the preconceptual period; (3) antenatal care, representing PHC during pregnancy; (4) skilled birth attendance, reflecting medical care at birth; and (5) postnatal care, representing health check-ups within the first 2 months of life (eFigure 1 and eAppendix 1.1 and eTable 1 and eAppendix 2.2 in Supplement 1).

To compute the chosen indicator of effective PHC coverage, each of the 5 constituent variables listed were transformed into a dichotomous format, classifying outcomes as either favorable or unfavorable. For the access variable, the absence of perceived barriers associated with distance and money was categorized as favorable. Family planning performed with modern methods was considered favorable.^5^ For antenatal care, a minimum of 4 visits during the antenatal period—essential for effective maternal health intervention—was deemed favorable.^5^ The presence of postnatal care within the 2 months after birth was also classified as favorable.^24^ Finally, skilled birth attendance was considered as favorable when there was a nurse, (auxiliary) midwife, or physician to support the delivery.^25,26^ The PHC index was the sum of the variables score for each individual. It was able to capture a gradient dimension, ranging from no service (no services and presence of access barriers) to low-intermediate service, intermediate service, high-intermediate service, and high service (all services received and absence of access barriers). As such, increasing index scores indicated a progressively stronger link of the child with PHC. Different PHC index specifications and scoring systems were analyzed and showed results consistent with the chosen one (eAppendix 2.2 in Supplement 1). This included a dichotomized version (ie, having no favorable variables vs having some favorable variables), separating the 2 perceived barriers into 2 variables, different subgrouping combinations of the variables analyzed, or analyzing the variables based on their chronological timeline of exposure.

### Covariates

We used a set of confounding variables—determinants of child mortality and potential confounders on the coverage and accessibility of PHC—at individual, household, and national levels to adjust the multivariable regression models. The covariate data were obtained from the same DHS datasets and from World Bank data.^20,27^ Covariates included the child’s sex and age, birth plurality, birth order, maternal age at first birth, maternal educational level, access to improved water or sanitation services, gross domestic product per capita based on purchasing power parity, the percentage of governmental gross domestic product allocated to health, the Gini index, and stunting prevalence (eTables 2-4 and eAppendix 2.3 and eTables 5-7 and eAppendix 2.4 in Supplement 1).

### Statistical Analysis

Statistical analysis was performed from October 2024 to May 2026. The association of effective PHC coverage with child mortality in SSA was assessed using a 2-way fixed-effects multivariable Poisson regression model^28^ with standard errors clustered at the child level (eAppendix 2.6 in Supplement 1). The models were weighted and adjusted for the confounding variables as well as for fixed effects by region within these 3 countries, as well as by year. The weight applied in the model was based on the survey design weight, standardized across surveys within each country, and further equalized across countries to ensure the equal contribution of each country (eAppendix 2.5 in Supplement 1). To verify the robustness of the results, a wide range of sensitivity analyses were conducted.^29^ Moreover, we developed 2 different triangulation analyses based on inverse probability of treatment weighting and propensity score matching analyses (eAppendix 3.3 in Supplement 1). To further analyze the possible causal interpretation of the results, we applied the framework of the Bradford Hill criteria for causal inference in epidemiology (eAppendix 3.4 in Supplement 1).^30^

A validated individual-level dynamic microsimulation model was used to estimate the association of different effective PHC coverage scenarios with child mortality, following methods from previous studies.^11,31^ A simulated cohort of children younger than 5 years was generated for each country using independent variables from the latest DHS survey. We performed 1000 Monte Carlo simulations to estimate each child’s mortality risk using a multivariable Poisson regression model, then aggregated survival outcomes to compute the mortality rate among children younger than 5 years by country and year. Scenarios were compared using rate ratios (RRs) and avoided deaths (eAppendices 4.2, 5.1, 5.2, 5.3, and 5.4 and eFigure 3 in Supplement 1).

The comparison spans from 2025 to 2035. The baseline scenario assumes that PHC index coverage remains unchanged. The defunding scenario begins with USAID funding cuts in 2025, followed by continued deterioration of PHC service until reaching no service (PHC index score = 0).^7^ The 2025 funding cuts correspond to PHC index reductions of 30.1% in Ethiopia, 7.8% in Gabon, and 55.7% in Mozambique, reflecting mean DAH contributions to health financing from 2014 to 2024 (eFigure 2, eTables 23 and 24, and eAppendix 4.1 in Supplement 1).^6^

The eMethods in Supplement 1 detail the forecasting method per the International Society for Pharmacoeconomics and Outcomes Research (ISPOR) and Society for Medical Decision Making (SMDM) reporting guidelines, alongside an algebraic deterministic analysis, with consistent results based on the microsimulations (eTable 29 and eAppendix 5.6 in Supplement 1).^32^ Analyses were performed with STATA, version 18 (StataCorp LLC) and R, version 4.3.1 (R Project for Statistical Computing).

## Results

**Figure 1.**
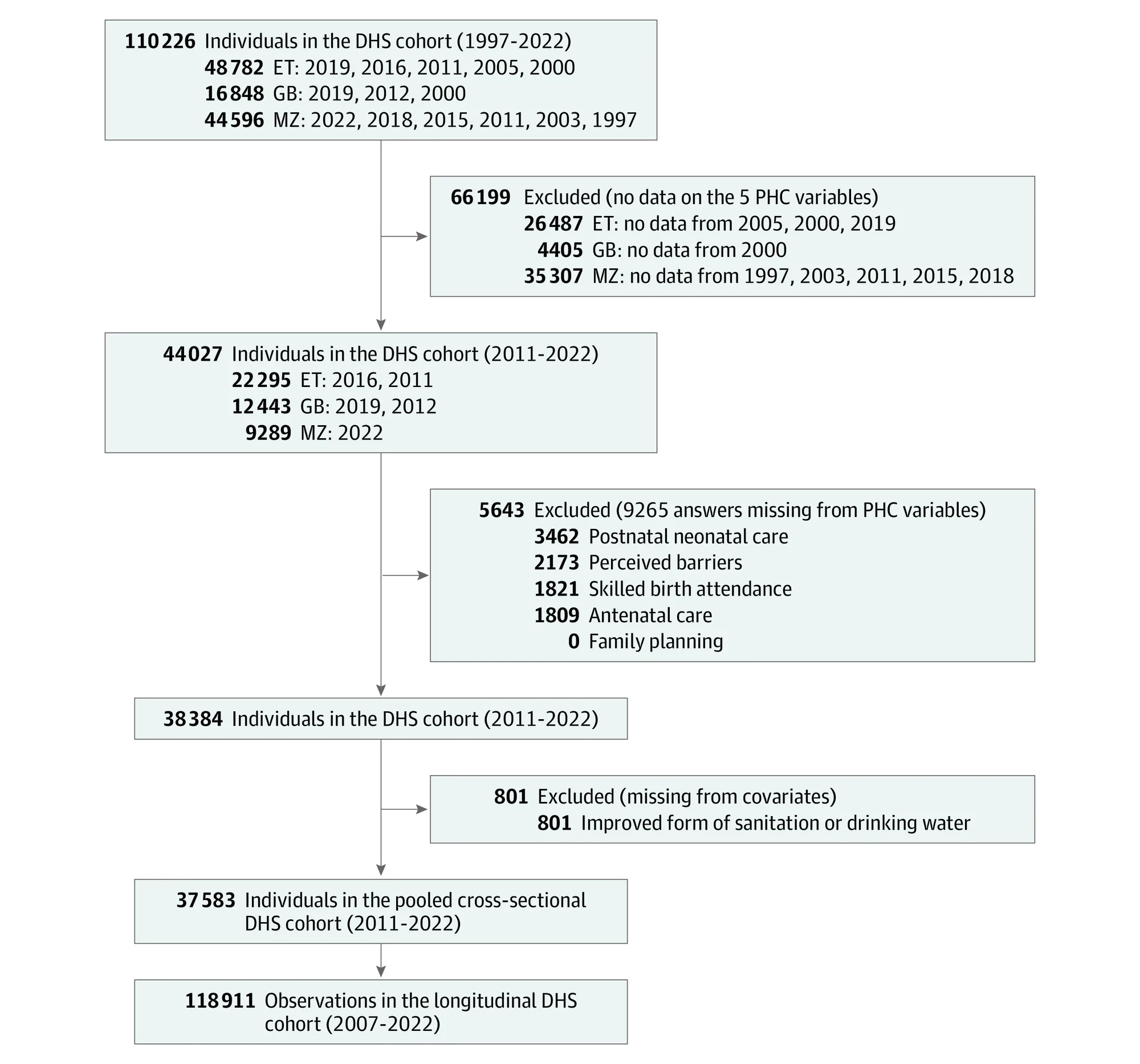
Flowchart of the Final Dataset DHS indicates Demographic and Health Surveys; ET, Ethiopia; GB, Gabon; MZ, Mozambique; and PHC, primary health care.

Figure 1 presents the sample selection flowchart. As shown, starting with all the standard DHS surveys, some DHS surveys were excluded due to the absence of data on PHC index variables. Among these, 5643 observations were removed due to missing PHC index data, and an additional 801 observations were discarded due to missing covariate information. Ultimately, the analysis included a total of 118 911 person-years, representing 37 583 children, drawn from 5 DHS surveys conducted between 2007 and 2022. Gabon and Ethiopia each had 2 surveys, accounting for 33 269 and 64 914 person-years, respectively, while Mozambique had 1 survey with 20 728 observations.

Table 1 shows the descriptive statistics of the sample. The analysis included 37 583 children younger than 5 years, equivalent to 118 911 child-years (mean [SD] age, 1.4 [1.3] years; 60 252 boys [50.7%] and 58 659 girls [49.3%]). Across these 118 911 child-year observations, there were 2166 deaths (1.8%). Ethiopia accounted for 2.1% of deaths (1338 of 64 914 child-years), Mozambique for 1.8% (364 of 20 728), and Gabon for 1.4% (464 of 33 269). Most deaths (1471 [67.9%]) were children younger than 1 year, of whom 1036 (70.4%) died before reaching the first month of life. The coverage by the individual PHC variables was consistently lower among the person-years of children who did not survive compared with those who did. The highest coverage was observed for skilled birth attendance (52 795 [44.4%]), followed by antenatal care (48 864 [41.1%]). In contrast, perceived barriers (29 044 [24.4%]), postnatal care (28 499 [24.0%]), and family planning (26 560 [22.3%]) had significantly lower coverage. Gabon had the greatest coverage of service provision, followed by Mozambique and Ethiopia, although Mozambique showed a marginally higher proportion in the highest category (Gabon, 796 of 33 269 [2.4%]); Mozambique, 554 of 20 728 [2.7%]; and Ethiopia, 317 of 64.914 [0.5%]); the inverse pattern was observed for the lowest coverage categories (Gabon, 2143 of 33 269 [6.4%]; Mozambique, 1976 of 20 728 [9.5%]; and Ethiopia, 32 189 of 64 914 [49.6%]).

**Table 1.** Descriptive Statistics of the Study Variables

Characteristic	Observations, No. (%)				
	Ethiopia	Mozambique	Gabon	Total
Child mortality, age^a^	64 914 (100)	20 728 (100)	33 269 (100)	118 911 (100)
<5 y	1338 (2.1)	364 (1.8)	464 (1.4)	2166 (1.8)
<1 y	919 (68.7)	261 (71.7)	291 (62.7)	1471 (67.9)
<1 mo	651 (70.8)	184 (70.5)	201 (69.1)	1036 (70.4)
1 y	248 (18.5)	65 (17.9)	105 (22.6)	418 (19.3)
2 y	111 (8.3)	23 (6.3)	53 (11.4)	187 (8.6)
3 y	42 (3.1)	9 (2.5)	8 (1.7)	59 (2.7)
4 y	18 (1.4)	6 (1.7)	7 (1.5)	31 (1.4)
PHC index variables	64 914 (100)	20 728 (100)	33 269 (100)	118 911 (100)
Family planning	14 247 (22.0)	5939 (28.7)	6374 (19.2)	26 560 (22.3)
Among deceased children	190 (14.2)	79 (21.7)	84 (18.1)	353 (16.3)
Among living children	14 057 (22.1)	5860 (28.8)	6290 (19.2)	26 207 (22.5)
Antenatal care	15 054 (23.2)	11 035 (53.2)	22 775 (68.5)	48 864 (41.1)
Among deceased children	236 (17.6)	191 (52.5)	282 (60.8)	709 (32.7)
Among living children	14 818 (23.3)	10 844 (53.3)	22 493 (68.6)	48 155 (41.3)
Skilled birth attendance	10 310 (15.9)	14 250 (68.8)	28 235 (84.9)	52 795 (44.4)
Among deceased children	152 (11.4)	14 005 (68.8)	364 (78.7)	762 (35.2)
Among living children	10 158 (16.0)	245 (67.3)	27 870 (85.0)	52 033 (44.6)
Postnatal care	4248 (6.5)	5552 (26.8)	18 699 (56.2)	28 499 (24.0)
Among deceased children	55 (4.1)	80 (22.0)	183 (39.4)	318 (14.7)
Among living children	4193 (6.6)	5472 (26.9)	18 516 (56.4)	28 181 (24.1)
No perceived barriers in distance to a health facility and/or money	13 579 (20.9)	9439 (45.5)	6026 (18.1)	29 044 (24.4)
Among deceased children	239 (17.9)	166 (45.6)	77 (16.6)	482 (22.3)
Among living children	13 340 (21.0)	9273 (45.5)	5949 (18.1)	28 562 (24.5)
Effective PHC index score	64 914 (100)	20 728 (100)	33 269 (100)	118 911 (100)
Covered by 0 services or access	32 189 (49.6)	1976 (9.5)	2143 (6.4)	36 308 (30.5)
Among deceased children	778 (58.2)	39 (10.7)	55 (11.9)	872 (40.3)
Among living children	31 411 (49.4)	1937 (9.5)	2088 (6.4)	35 436 (30.4)
Covered by 1 service or access	17 683 (27.2)	4020 (19.4)	4062 (12.2)	25 765 (21.7)
Among deceased children	344 (25.7)	72 (19.8)	76 (16.4)	492 (22.7)
Among living children	17 339 (27.3)	3948 (19.4)	3986 (12.2)	25 273 (21.7)
Covered by 2 services or access	8146 (12.6)	5912 (28.5)	9616 (28.9)	23 674 (19.9)
Among deceased children	135 (10.1)	112 (30.8)	146 (31.5)	393 (18.1)
Among living children	8011 (12.6)	5800 (28.5)	9470 (28.9)	23 281 (19.9)
Covered by 3 services or access	4438 (6.8)	5463 (26.4)	11 773 (35.4)	21 674 (18.2)
Among deceased children	66 (4.9)	103 (28.3)	132 (28.5)	301 (13.9)
Among living children	4372 (6.9)	5360 (26.3)	11 641 (35.5)	21 373 (18.3)
Covered by 4 services or access	2141 (3.3)	2803 (13.5)	4879 (14.7)	9823(8.3)
Among deceased children	15 (1.1)	34 (9.3)	48 (10.3)	97 (4.5)
Among living children	2126 (3.3)	2769 (13.6)	4831 (14.7)	9726 (8.3)
Covered by 5 services or access	317 (0.5)	554 (2.7)	796 (2.4)	1667(1.4)
Among deceased children	0	4 (1.1)	7 (1.5)	11 (0.5)
Among living children	317 (0.5)	550 (2.7)	789 (2.4)	1656 (1.4)
Covariates	64 914 (100)	20 728 (100)	33 269 (100)	118 911 (100)
Male child	33 454 (51.5)	10 147 (49.0)	16 651 (50.1)	60 252 (50.7)
Female child	31 460 (48.5)	10 581 (51.0)	16 618 (49.9)	58 659 (49.3)
Child’s age, mean (SD), y	1.4 (1.3)	1.2 (1.2)	1.5 (1.3)	1.4 (1.3)
Child is a twin	1569 (2.4)	672 (3.2)	1203 (3.6)	3444 (2.9)
Birth order (first child or second or third child)	20 366 (31.4)	7900 (38.1)	12 266 (36.9)	40 532 (34.1)
Birth order (first vs fourth or more children)	32 836 (50.6)	7436 (35.9)	13 135 (39.5)	53 407 (44.9)
Maternal age at first birth >18 y	38 925 (60.0)	10 443 (50.4)	19 291 (58.0)	68 659 (57.7)
Maternal educational level (no or primary education vs secondary or higher)	3696 (5.7)	4349 (21.0)	20 032 (60.2)	28 077 (23.6)
Improved form of sanitation and/or drinking water	37 464 (57.7)	13 500 (65.1)	26 652 (80.1)	77 616 (65.3)
GDP per capita in PPP, mean (SD), USD	1612.20 (320.34)	1480.23 (26.08)	19 002.94 (544.24)	6453.79 (7829.95)
Governmental GDP spending on health, mean (SD), %	4.3 (0.6)	8.5 (0.6)	2.8 (0.4)	4.6 (2.0)
Gini coefficient, mean (SD)	33.3 (1.3)	50.3 (0.2)	39.1 (1.5)	37.9 (6.4)
National percentage of stunting, mean (SD)	43.1 (3.3)	37.4 (0.7)	16.2 (2.1)	34.6 (11.9)
Total No. of observations	64 914	20 728	33 269	118 911
No. of surveys	2	1	2	5

Abbreviations: GDP, gross domestic product; PHC, primary health care; PPP, purchasing power parity.

^a^ *Child mortality* refers to the person-year dyad in which a child died.

The obstetric variables showed that most mothers gave birth for the first time when they were older than 18 years (68 659 [57.7%]), and most commonly gave birth to 4 or more children (53 407 [44.9%]) (Table 1). The sociodemographic variables suggested that only a minority of mothers (28 077 [23.6%]) had secondary or higher education, while 77 616 (65.3%) had access to an improved source of drinking water or improved sanitation. Socioeconomic variables varied across countries, with Gabon showing the most favorable indicators, followed by Mozambique, while Ethiopia ranked the lowest (eTables 8A and 8B and eAppendix 3.1 in Supplement 1).

Table 2 shows the results of the different Poisson regression models. The final adjusted model (PHC-4), including all fixed effects, individual variables, and aggregate variables, showed a significant association between PHC and reduced child mortality. The low PHC index category was associated with a 17% mortality risk reduction (RR, 0.83 [95% CI, 0.69-0.99]). The intermediate PHC index level was associated with a 23% risk reduction (RR, 0.77 [95% CI, 0.60-0.99]). The high intermediate PHC index level was associated with a 36% risk reduction (RR, 0.64 [95% CI, 0.46-0.90]), and the high PHC index level was associated with a 58% risk reduction (RR, 0.42 [95% CI, 0.19-0.91]). The low intermediate PHC index category was not significant, showing a similar risk reduction to the low category. Stratification analysis by country was also conducted, and the results were consistent with the main findings (eTables 9-13 and eAppendix 3.2 in Supplement 1). All sensitivity and triangulation analyses supported the results.

**Table 2.** Poisson Regression Models^a^

Variables	Child mortality, RR (95% CI)			
	Model PHC-1	Model PHC-2	Model PHC-3	Model PHC-4
PHC index score				
PHC index (no service and access)	1.00 [Reference]	1.00 [Reference]	1.00 [Reference]	1.00 [Reference]
PHC index (low)	0.79 (0.66-0.95)	0.84 (0.70-1.01)	0.82 (0.69-0.99)	0.83 (0.69-0.99)
PHC index (low intermediate)	0.77 (0.64-0.92)	0.87 (0.70-1.07)	0.81 (0.65-1.00)	0.83 (0.67-1.03)
PHC index (intermediate)	0.65 (0.54-0.80)	0.79 (0.61-1.01)	0.74 (0.58-0.94)	0.77 (0.60-0.99)
PHC index (high intermediate)	0.51 (0.384-0.69)	0.62 (0.45-0.87)	0.60 (0.43-0.83)	0.64 (0.46-0.90)
PHC index (high)	0.32 (0.15-0.68)	0.39 (0.18-0.84)	0.39 (0.18-0.84)	0.42 (0.19-0.91)
Region and year fixed effects	No	Yes	Yes	Yes

Abbreviations: PHC, primary health care; RR, rate ratio.

^a^ Crude model (PHC-1) purely covers the association between the PHC index score and vital status. PHC-2 adds fixed effects for year and region. PHC-3 adds demographic and obstetric covariates. PHC-4 adds socioeconomic and country level covariates. Analysis included children younger than 5 years (n = 37 583), leading to 118 911 person-years.

**Figure 2.**
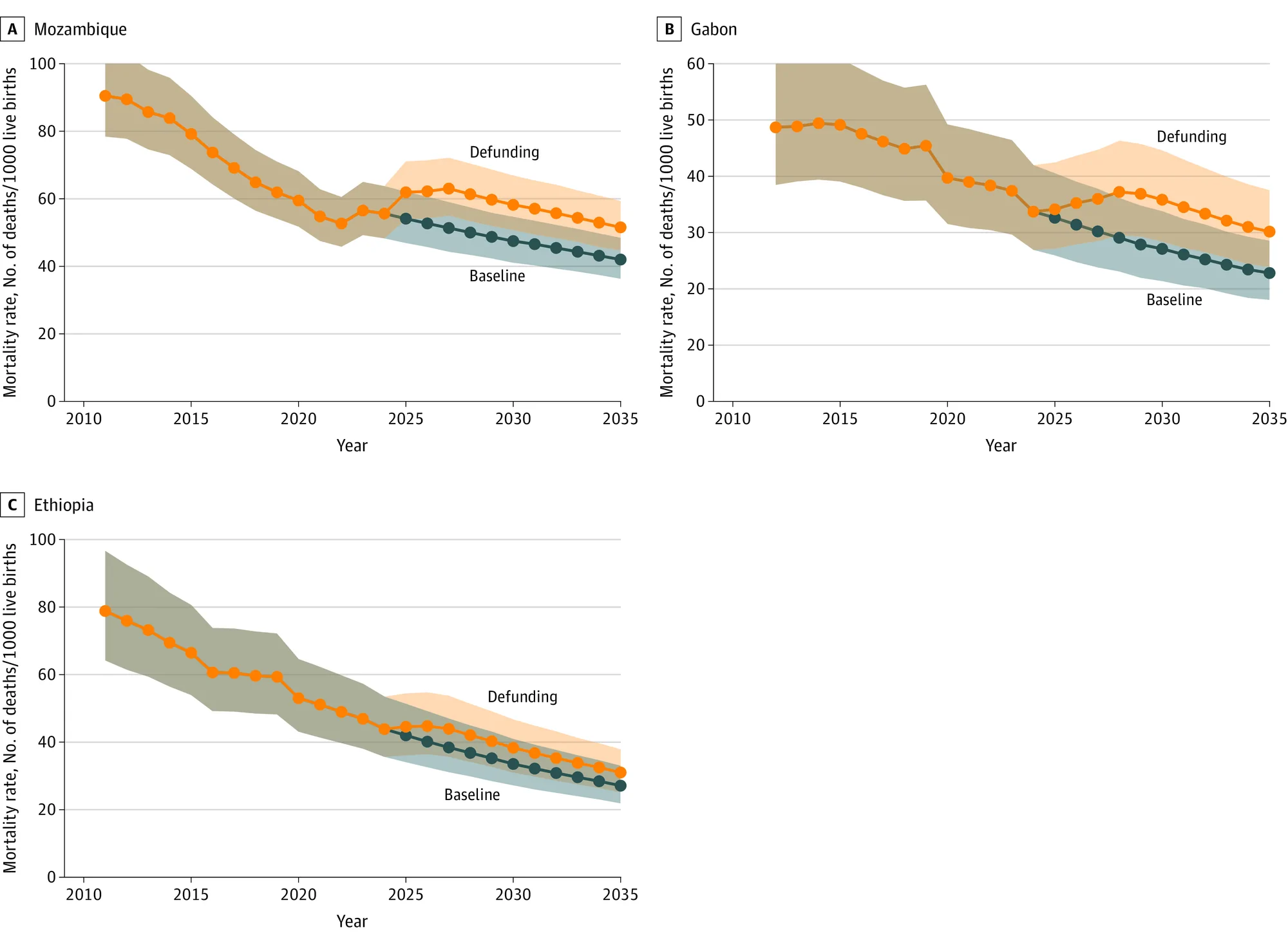
Line Graphs Showing Estimated Mortality Rates Among Children Younger Than 5 Years (Number of Deaths by 1000 Live Births) Under the Baseline Scenario and Defunding Scenario for Mozambique, Gabon, and Ethiopia Up to 2035 Shaded areas indicate 95% uncertainty intervals.

Figure 2 shows the forecasted evolution of the mortality rate among children younger than 5 years for the 3 countries across time for each scenario until 2035, showing that PHC deterioration is associated with increased mortality rates. Table 3 shows the resulting RRs between the baseline scenario and defunding scenario for each country, plus the total preventable deaths in the period of analysis. The results show that the mortality rate among children younger than 5 years in the baseline scenario in comparison with the defunding scenario, for 2035, would be 18.5% lower for Mozambique (RR, 0.82; 95% uncertainty interval [UI], 0.80-0.83), 24.4% lower for Gabon (RR, 0.76; 95% UI, 0.73-0.78), and 12.6% lower for Ethiopia (0.87; 95% UI, 0.87-0.88). The number of preventable child deaths in all 3 countries would total an estimated 373 108 (95% UI, 313 152-444 244): 156 082 (95% UI, 136 347-178 560) in Mozambique, 5511 (95% UI, 4379-6871) in Gabon, and 211 515 (95% UI, 172 426-258 813) in Ethiopia across the 2025 to 2035 period (eTables 26-28 and eAppendix 5.5 in Supplement 1).

**Table 3.** RRs and Number of Preventable Deaths Between the Baseline and Defunding Scenarios

Year	Baseline vs defunding scenarios, RR (95% UI)		
	Mozambique	Gabon	Ethiopia
2025	0.87 (0.87-0.88)	0.96 (0.95-0.97)	0.94 (0.94-0.95)
2030	0.82 (0.80-0.83)	0.76 (0.74-0.78)	0.88 (0.87-0.88)
2035	0.82 (0.80-0.83)	0.76 (0.73-0.78)	0.87 (0.87-0.88)
Total No. of preventable deaths (95% UI), 2025-2035	156 082 (136 347-178 560)	5511 (4379-6871)	211 515 (172 426-258 813)

Abbreviations: RR, rate ratio; UI, uncertainty interval.

## Discussion

To our knowledge, this is the first study to comprehensively evaluate the association of PHC with child mortality in SSA, analyzing nearly 120 000 person-years over almost 2 decades. It also added a forecasting analysis of the potential associations of dismantling PHC coverage, triggered by the current DAH crisis, with child mortality up to 2035. The study’s main findings suggest a significant association of PHC coverage with a reduction in the mortality rate among children younger than 5 years during the past 2 decades in Mozambique, Gabon, and Ethiopia. As the PHC expands to encompass additional services or improves its access, the reduction becomes larger, with a dose-response association that reaches mortality reductions of 58% with the highest coverage. Our forecasting provides evidence that dismantling of PHC coverage, which could occur due to DAH funding cuts, could be associated with more than 373 000 preventable child deaths across the 3 countries between 2025 and 2035.

Although no research has directly examined the association of PHC with child mortality in SSA at the multicountry level, there is evidence on the efficacy of specific PHC services across different child age subgroups in SSA, aligned with the findings of our study. Interventions such as family planning, antenatal care, skilled birth attendance, and postnatal care have been shown to have significant protective associations with mortality across different SSA countries.^14,15,16,17,18,19^ Furthermore, our results are in line with research conducted in the Latin American region based on aggregate-level data and are a nominal indicator of PHC coverage. A study in different Latin American countries found a mean 18.6% protective effect on the mortality rate among children younger than 5 years under full PHC coverage,^11^ while another study focused on infants in Brazil found that a 10% increase in expansion of the national PHC program was able to reduce mortality by 4.5%.^12^

Although effective PHC coverage has been enhanced in SSA in recent years, further improvements could be associated with greater reductions in child mortality. Ethiopia has been expanding its PHC level network for the past 2 decades through the building up of integrated PHC health services at the district level and the deployment of community health workers at the village level.^33^ Mozambique has been strengthening its PHC system through the significant expansion of their technical workforce within health services and among community health workers.^34,35^ Gabon—characterized by a hospital-centered PHC system—is making efforts to integrate community health workers (originally deployed due to COVID-19 contingencies) to its health care system.^36^ The 3 countries have made progress in PHC delivery through the expansion of community-based health care. Many of the services captured in the index—as well as broader health outcomes, for which it serves as a proxy—can be further strengthened through PHC expansion at the community level, particularly by reaching rural and underserved populations where facility-based care remains limited. Community health workers represent a key strategy to bridge the gap between formal health systems and communities, with demonstrated potential to reduce child mortality from preventable causes, such as pneumonia, diarrhea, and malaria.^37,38,39,40^

Although this study underscores the need to continue advancing with further provisions and better access to PHC services, it also points out that this advancement may be currently jeopardized, as SSA has started to experience substantial reductions in international financial support, primarily due to policy shifts and budgetary constraints in donor nations, which may significantly affect preventable child deaths in the upcoming years.^6,7,8^

### Strengths and Limitations

This study has some strengths. First, the integration of a large, retrospective longitudinal, multicountry analysis with microsimulation forecasting models enhances the reliability and accuracy of our projections. Second, the use of individual-level data for exposure, outcomes, and the large set of covariates—with the inclusion of higher-level variables and 2-way subregional and time-fixed effects—strengthens the accuracy of our estimates, reduces the possibility of biases, and rules out any ecologic fallacy, one of the main limitations of previous studies. Third, our study focuses on effective coverage rather than nominal coverage, as in most previous research, thereby capturing the actual accessibility and use of qualified health care services.

This study also has some limitations, including the inherent challenge of comprehensively measuring PHC. Various frameworks, such as the WHO Primary Health Care Performance Initiative conceptual framework,^22^ have been developed to systematize this measurement. However, even these frameworks highlight the breadth and complexity of PHC, making it difficult to be quantified accurately, especially in a multicountry analysis. Despite this limitation, we developed a well-rounded, cross-country comparable variable set by incorporating diverse, representative, and closely linked aspects of the association of families with the provision and access of integrated PHC services. Another limitation of this study includes challenges associated with data availability regarding the individual variables included in the PHC index. As a result, some variables that may have been suitable as proxies of effective PHC coverage had to be discarded. Despite this, we were able to include a sample of variables that robustly covered the fundamental, internationally recognized PHC services. Further sensitivity analyses demonstrated that the use of different indicator specifications showed consistent results with the main findings (eAppendix 3.2 in Supplement 1).

A further limitation lies in the fact that our study had an observational design and did not use a quasi-experimental approach, which is regarded as a higher standard for causal inference, because such an approach would not have allowed us to analyze a dose-response effect. Despite this, we conducted multiple triangulation analyses using distinct quasi-experimental approaches, and the consistency of these results with our main findings, together with the adherence to the Bradford Hill criteria, strengthened the confidence that the statistical associations may represent true causal relationships (eTable 22 and eAppendix 3.4 in Supplement 1).

Another limitation is that the forecasted scenarios carry inherent uncertainties and assumptions. It is difficult to estimate the extent of the associations of decreased DAH with effective PHC coverage in the future or how national governments may react to mitigate decreased DAH, as well as other exogenous shocks that could occur and affect PHC. For instance, there is already evidence of governments responding to the DAH crisis, although these efforts may be insufficient.^41^ However, the main aim of the forecasting is to do a comparison against a worst-case scenario rather than a perfect estimation, thus, to compare 2 parallel scenarios that differ only in the path of effective PHC coverage. Given that, mortality RRs and differences in the number of deaths are expected to remain valid and robust.

## Conclusions

In this cohort study, we found evidence that effective PHC coverage is associated with reduced child mortality in SSA. Strengthening and expanding PHC would be essential for advancing progress toward child-related Sustainable Development Goals, while our findings also underscore the potentially severe consequences that ongoing reductions in DAH may have on mortality among children younger than 5 years in the future.

## Notes

Supplement 1. **eMethods 1.** Retrospective Analysis **eAppendix 1.1.** Primary Health Care Conceptual Framework **eFigure 1.** Study Conceptual Framework **eAppendix 1.2.** Primary Health Care Systems **eAppendix 1.3.** Literature Search **eAppendix 2.1.** Transformation to a Longitudinal Dataset **eAppendix 2.2.** Selection Procedure of the PHC Variables **eTable 1.** DHS Variables Analyzed for Composing the PHC-Index **eAppendix 2.3.** Categorization of the Covariates **eTable 2.** Representing the Covariates Used in the Data Analysis, Including Their DHS Coding and the Labels Attached to Them **eTable 3.** Green Represents Improved Drinking Water, White Represents Unimproved Drinking Water and Orange Represents Missing **eTable 4.** Green Represents Improved Sanitation, White Represents Unimproved Sanitation and Orange Represents Missing **eAppendix 2.4.** Interpolation and Extrapolation Method **eTable 5.** Representing the Country Level Data in Mozambique **eTable 6.** Representing the Country Level Data in Ethiopia **eTable 7.** Representing the Country Level Data in Gabon **eAppendix 2.5.** Different Weights Analyzed **eAppendix 2.6**. Equation **eAppendix 3.1.** Descriptives **eTable 8A.** Descriptive Statistics of the Study Variables **eTable 8B.** Descriptive Statistics of the Study Variables Across Time **eAppendix 3.2.** Stratification Analyses **eTable 9.** Poisson Regression Models Stratified by Sex Including the Categories Female and Male **eTable 10.** Poisson Regression Models Stratified by Age Including the Categories, Age Below 1 Year and 1 to 4 Years **eTable 11.** Poisson Regression Models Stratified by Area of Residence Including the Categories, Rural and Urban **eTable 12.** Poisson Regression Models Stratified by Wealth Index Including the Categories, Poorest/Poorer and Middle/Richer/Richest **eTable 13.** Poisson Regression Models Stratified by Country Including The Categories, Ethiopia, Gabon and Mozambique **eAppendix 3.3.** Sensitivity and Triangulation Analyses **eTable 14.** Different Potential Primary Health Care (PHC) Index Scores **eTable 15.** Poisson Regression Models Based on Different Weights, Starting With the Original Survey Design Weight, Then Adjusted by Country, Followed by Only an Adjustment by Survey and Finally by Country and Survey **eTable 16.** Poisson Regression Model Versus Logistic Regression Model Including the Same Covariates, Fixed Effects and Adjustment by Weight **eTable 17.** Poisson Regression Models Based on Different Weights, Starting With the Original Survey Design Weight Divided by the Number of Follow-Up Years, Then Adjusted by Country, Followed by Only an Adjustment by Survey and Finally by Country and Survey **eTable 18.** Poisson Regression Model for the Longitudinal Dataset Versus Poisson Regression Model for the Pooled Cross-Sectional Dataset **eTable 19.** Logistic Regression Model for the Longitudinal Dataset Versus Logistic Regression Model for the Pooled Cross-Sectional Dataset **eTable 20.** Crude Model (PHC-1) on the Left Side of the Table, Purely Covering the Impact Between the Primary Health Care Index Score and Vital Status, Followed by Adding Fixed Effects for Year and Region (PHC-2), Followed by the Addition of the Demographic and Obstetric Covariates (PHC-3) and Finalizing With the Addition of the Socioeconomic and Country Level Covariates (PHC-4) **eTable 21.** PHC-4 for Which Analysis Included Children Under-Five Years of Age (n=37,583) Leading to 118,911 Observations; PHC_2 for the IPTW and PSM Triangulation **eAppendix 3.4.** Bradford Hill Causality Criteria **eTable 22.** Bradford Hill Causality Criteria **eMethods 2.** Forecasting Analysis **eAppendix 4.1.** Scenarios Contextual Background **eFigure 2.** Bar Graphs on Data About Spending Sources % Over Total Health Spending by Analyzed Country, Period 2014-2024 **eTable 23.** DAH % as of Total Health Spending by Analyzed Country, 2014-2024 **eTable 24.** DAH % as of Total Health Spending (Minimum, Maximum and Average) by Analyzed Country, 2014-2024 **eAppendix 4.2.** Dataset **eAppendix 5.1.** Description of the Forecasting Methodology **eAppendix 5.2.** Purpose of the Forecasting and its Applications **eAppendix 5.3.** First Stage: Synthetic Cohort Creation **eTable 25.** Under-Five Population and Under-Five Deaths for 2011 Ethiopia, 2011 Mozambique and 2012 Gabon Sub-Cohorts, by Age Group **eAppendix 5.4.** Second Stage: Prediction Methodology **eFigure 3.** Validation Data and Graphs, Including U5MR`s (95%UI) Resulted From the Modelling and IGME U5MR’s, Period 2011/12 to 2030 **eAppendix 5.5.** Results **eTable 26.** Descriptive PHC Synthetic Cohort Statistics, Baseline and Defunding Scenarios **eTable 27.** Descriptive Statistics of the PHC-Index by Scenario (Proportion of Under-Five Children in the PHC-Index Category) **eTable 28.** Averted Deaths (95%UI) (AD) in Baseline vs Defunding Scenario, by Year and Country **eAppendix 5.6.** Triangulation Analysis **eTable 29.** Preventable Fraction of the Population Analysis
eReferences.

Supplement 2.
Data Sharing Statement
