# Errors in Figure Labels

**DOI:** 10.1001/jamanetworkopen.2026.39490

**Published:** 2026-09-22

In the Original Investigation titled, “Primary Health Care Coverage and Child Mortality in Sub-Saharan Africa,”^1^ published August 7, 2026, there were errors in the Figure 2 y-axis labels. The labels have been changed from “Mortality rate, %” to “Mortality rate, No. of deaths/1000 live births.” This article has been corrected.^1^
