# Supplementary material for: Primary Health Care Coverage and Child Mortality in Sub-Saharan Africa
Source: JAMA Netw Open. 2026 Aug 7;9(8):e2627655. doi: 10.1001/jamanetworkopen.2026.27655 (PMC13451977; doi:10.1001/jamanetworkopen.2026.27655)
Supplement: Supplementary file 1 [file jamanetwopen-e2627655-s001.pdf]

Supplementary Online Content

Barreix G, ter Braak S, Albino Pena D, et al. Primary health care coverage and child mortality in sub-Saharan Africa. *JAMA Netw Open.* 2026;9(8):e2627655. doi:10.1001/jamanetworkopen.2026.27655

eMethods 1. Retrospective Analysis
eAppendix 1.1. Primary Health Care Conceptual Framework
eFigure 1. Study Conceptual Framework
eAppendix 1.2. Primary Health Care Systems
eAppendix 1.3. Literature Search
eAppendix 2.1. Transformation to a Longitudinal Dataset
eAppendix 2.2. Selection Procedure of the PHC Variables
eTable 1. DHS Variables Analyzed for Composing the PHC-Index
eAppendix 2.3. Categorization of the Covariates
eTable 2. Representing the Covariates Used in the Data Analysis, Including Their DHS Coding and the Labels Attached to Them
eTable 3. Green Represents Improved Drinking Water, White Represents Unimproved Drinking Water and Orange Represents Missing
eTable 4. Green Represents Improved Sanitation, White Represents Unimproved Sanitation and Orange Represents Missing
eAppendix 2.4. Interpolation and Extrapolation Method
eTable 5. Representing the Country Level Data in Mozambique
eTable 6. Representing the Country Level Data in Ethiopia
eTable 7. Representing the Country Level Data in Gabon
eAppendix 2.5. Different Weights Analyzed
eAppendix 2.6. Equation
eAppendix 3.1. Descriptives
eTable 8A. Descriptive Statistics of the Study Variables
eTable 8B. Descriptive Statistics of the Study Variables Across Time
eAppendix 3.2. Stratification Analyses
eTable 9. Poisson Regression Models Stratified by Sex Including the Categories Female and Male
eTable 10. Poisson Regression Models Stratified by Age Including the Categories, Age Below 1 Year and 1 to 4 Years
eTable 11. Poisson Regression Models Stratified by Area of Residence Including the Categories, Rural and Urban
eTable 12. Poisson Regression Models Stratified by Wealth Index Including the Categories, Poorest/Poorer and Middle/Richer/Richest
eTable 13. Poisson Regression Models Stratified by Country Including The Categories, Ethiopia, Gabon and Mozambique
eAppendix 3.3. Sensitivity and Triangulation Analyses
eTable 14. Different Potential Primary Health Care (PHC) Index Scores

© 2026 Barreix G et al. *JAMA Network Open.*

eTable 15. Poisson Regression Models Based on Different Weights, Starting With the Original Survey Design Weight, Then Adjusted by Country, Followed by Only an Adjustment by Survey and Finally by Country and Survey
eTable 16. Poisson Regression Model Versus Logistic Regression Model Including the Same Covariates, Fixed Effects and Adjustment by Weight
eTable 17. Poisson Regression Models Based on Different Weights, Starting With the Original Survey Design Weight Divided by the Number of Follow-Up Years, Then Adjusted by Country, Followed by Only an Adjustment by Survey and Finally by Country and Survey
eTable 18. Poisson Regression Model for the Longitudinal Dataset Versus Poisson Regression Model for the Pooled Cross-Sectional Dataset
eTable 19. Logistic Regression Model for the Longitudinal Dataset Versus Logistic Regression Model for the Pooled Cross-Sectional Dataset
eTable 20. Crude Model (PHC-1) on the Left Side of the Table, Purely Covering the Impact Between the Primary Health Care Index Score and Vital Status, Followed by Adding Fixed Effects for Year and Region (PHC-2), Followed by the Addition of the Demographic and Obstetric Covariates (PHC-3) and Finalizing With the Addition of the Socioeconomic and Country Level Covariates (PHC-4)
eTable 21. PHC-4 for Which Analysis Included Children Under-Five Years of Age (n=37,583) Leading to 118,911 Observations; PHC_2 for the IPTW and PSM Triangulation
eAppendix 3.4. Bradford Hill Causality Criteria
eTable 22. Bradford Hill Causality Criteria
eMethods 2. Forecasting Analysis
eAppendix 4.1. Scenarios Contextual Background
eFigure 2. Bar Graphs on Data About Spending Sources % Over Total Health Spending by Analyzed Country, Period 2014-2024
eTable 23. DAH % as of Total Health Spending by Analyzed Country, 2014-2024
eTable 24. DAH % as of Total Health Spending (Minimum, Maximum and Average) by Analyzed Country, 2014-2024
eAppendix 4.2. Dataset
eAppendix 5.1. Description of the Forecasting Methodology
eAppendix 5.2. Purpose of the Forecasting and its Applications
eAppendix 5.3. First Stage: Synthetic Cohort Creation
eTable 25. Under-Five Population and Under-Five Deaths for 2011 Ethiopia, 2011 Mozambique and 2012 Gabon Sub-Cohorts, by Age Group
eAppendix 5.4. Second Stage: Prediction Methodology
eFigure 3. Validation Data and Graphs, Including U5MR`s (95%UI) Resulted From the Modelling and IGME U5MR's, Period 2011/12 to 2030
eAppendix 5.5. Results
eTable 26. Descriptive PHC Synthetic Cohort Statistics, Baseline and Defunding Scenarios
eTable 27. Descriptive Statistics of the PHC-Index by Scenario (Proportion of Under-Five Children in the PHC-Index Category)
eTable 28. Averted Deaths (95%UI) (AD) in Baseline vs Defunding Scenario, by Year and Country
eAppendix 5.6. Triangulation Analysis
eTable 29. Preventable Fraction of the Population Analysis
eReferences.

This supplementary material has been provided by the authors to give readers additional information about their work.

© 2026 Barreix G et al. *JAMA Network Open.*

eMethods 1. Retrospective Analysis

Part I – Background on Primary Health Care in the Countries Under Study

eAppendix 1.1. Primary Health Care Conceptual Framework

Malaria, congenital birth disorders, neonatal conditions, and lower respiratory infections are among the leading contributors to the child disease burden in the Sub-Saharan (SSA) region, significantly impacting under-five mortality rates (U5MR). [1]

One of the key social determinants of health, playing a crucial role in mitigating this burden and reducing child mortality, is the Primary Health Care (PHC) system. Extensive evidence has demonstrated the beneficial effects of PHC on various health outcomes, particularly child health[2]. PHC serves as the entry point to the healthcare system and encompasses a range of functions, including health promotion, disease prevention, and curative services.[3]

PHC services can be delivered through a facility-based approach or and complemented by a community-based strategy (see eFigure 1). In facility-based PHC, healthcare facilities such as hospitals, health centers, and posts are responsible for service provision, whereas in community-based PHC, community healthcare workers (CHWs) play a central role in delivering healthcare at the community level. [4] A well-integrated PHC system incorporates both approaches, ensuring comprehensive service delivery and maximizing population coverage. [5]

The effective coverage of PHC services in SSA is largely influenced by accessibility, which includes financial, geographical, and other barriers.[6–9] Studies have consistently shown that obstacles to healthcare access reduce the effectiveness of PHC services, thereby limiting their impact on health outcomes (see eFigure 1). Among the essential services provided through PHC, family planning (FP), antenatal care (ANC), skilled birth attendance (SBA), and postnatal care (PNC) are instrumental in reducing child morbidity and mortality. The availability and accessibility of these services across the continuum of care, along with the absence of access barriers, are critical in ensuring effective PHC coverage. As many of these services are covering a child -and along the nonexistence of access barriers- not only denote a more comprehensive PHC effective coverage but at the same time it proxies in a more confident way that is being covered by the PHC system in an integral, continuous and structural sense- regardless of the precise definition of PHC utilized (see eFigure 1).

© 2026 Barreix G et al. *JAMA Network Open.*

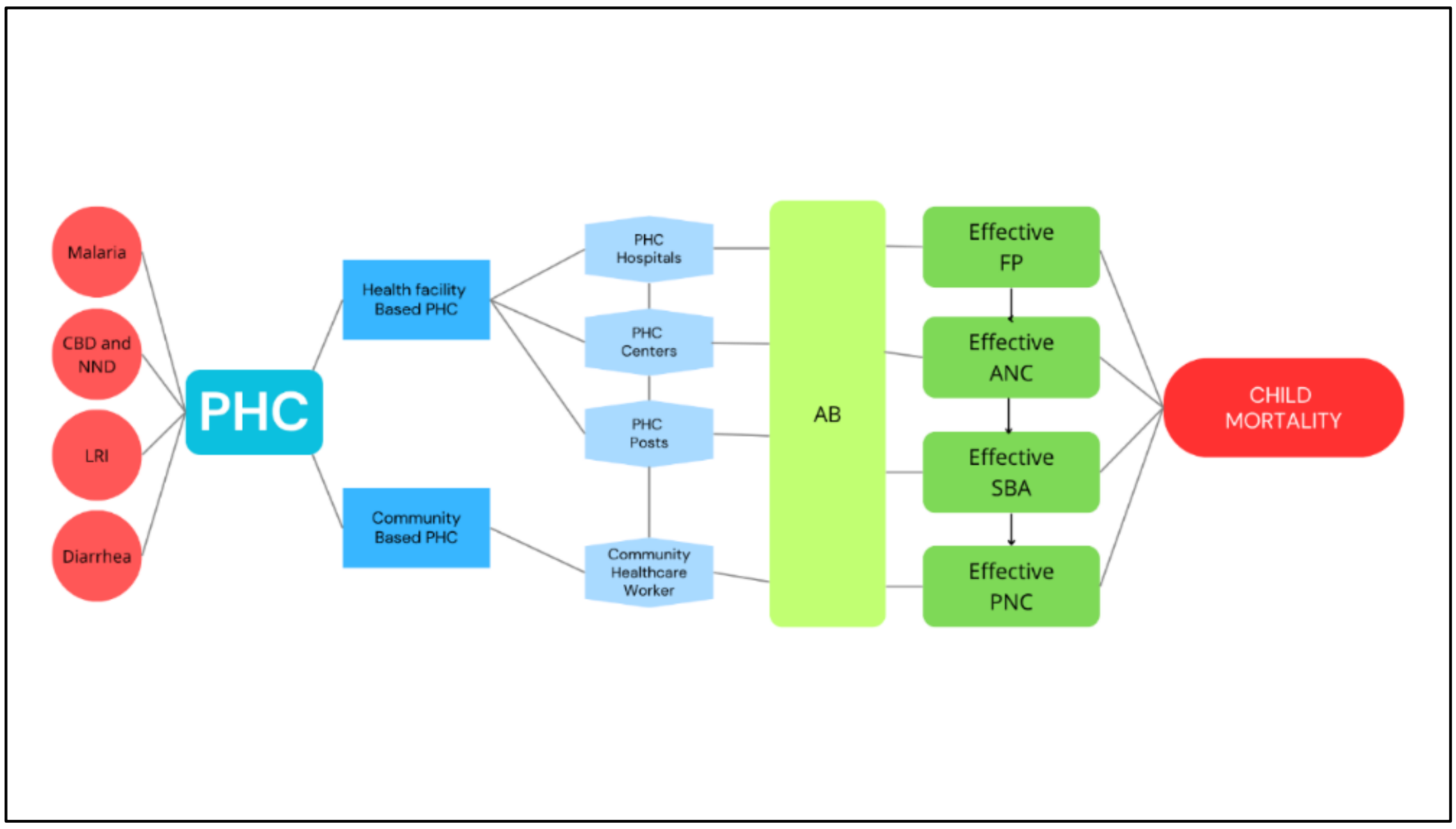

eFigure 1. Study Conceptual Framework. Note: CBD= congenital birth disorders, NND= neonatal disorders, LRI= lower respiratory infections, PHC= primary healthcare, AB= access barriers, FP= family planning, ANC= antenatal care; SBA= skilled birth attendance, PNC= postnatal care, Source: own generation

eAppendix 1.2. Primary Health Care Systems

1.2.1: PRIMARY HEALTH CARE - ETHIOPIA

PHC coverage in Ethiopia is defined as the proportion of the population covered by PHC services within 2 hours walking distance.[10] The Ethiopian PHC structure is built upon primary hospitals, health centers and health posts. Health posts are the first point of contact for rural populations and are usually staffed by Health Extension Workers. These are trained to deliver essential health services such as maternal and child health care, FP, health education and immunizations, and belong to the CHW´s network.[11] Each health post serves a catchment area of approximately 5000 people. [12]

Health centers are the second level of PHC in Ethiopia, usually located in more urbanized areas or administrative centers.[13] Health centers offer more advanced services than health posts, thereby functioning as a referral for health posts by providing curative and preventive care.[13] They typically serve a population of approximately 25000 people.[12] The primary hospitals serve 60000-100000 people and primarily provide emergency surgical services such as caesarean sections and blood transfusion services.[12]

In 2015, the Ethiopian government implemented a five-year plan called the Health Sector Transformation Plan I (HSTP-I) to reform and strengthen the country's health system.[12] This plan aimed to expand access to basic health services through community-based approaches, to strengthen the health workforce by improving the distribution and training of health personnel, to enhance health infrastructure by increasing the capacity of health facilities, and to improve service delivery standards. Additionally, it sought to increase domestic health financing and ensure its financial sustainability.[12]

Building on this progress, the Ethiopian government launched the Health Sector Transformation Plan II (HSTP-II) in 2020.[14] This new plan focused on more concrete objectives, including ensuring the provision of qualified PHC services, strengthening health information systems, addressing non-communicable diseases, implementing universal health insurance, and improving health system governance by ensuring transparency and reducing inefficiencies in service delivery.[14]

These transformation plans demonstrate Ethiopia's commitment to enhancing its health care system, promoting health equity, and achieving long-term health goals such as Universal Health Coverage (UHC) and improved health outcomes for all Ethiopians.

1.2.2: PRIMARY HEALTH CARE - GABON

The Gabonese health care system is divided into three main sectors, namely the public health care sector covering civilians and the military, the para-public health care sector created by providers of the National Social Security Fund and the private health care sector including profit, non-profit and traditional medicine.[15] The public health care sector can be further divided into primary (peripheral), secondary (intermediate) and tertiary (central) level.[15] The PHC sector consists of dispensaries and health posts at a community level, health centers in more urban areas and departmental hospitals functioning as the reference facility for PHC at the departmental level.[7] Based on data from 2018, this PHC system was composed of 47 departmental hospitals, 34 health centers, 413 dispensaries and 157 health posts.[15]

The services offered by the health posts include ANC, delivery care, PNC, vaccination, infant growth monitoring and FP of which ANC, delivery care, vaccination and postpartum care are most commonly reached out to.[8] The care covered by dispensaries is very limited, since many are closed or do not meet the required standards.[6]

Gabon was the first SSA country that created an obligatory national health insurance for everyone by January 4 2007, thereby also including the poorest portion of the population.[15] The goal was to increase health equity by reducing the out-of-pocket health expenditure, which decreased substantially from 50% in 2007 to 21.77% by now.[16] Further commitment was shown by the National Health strategy launched in 2010, which has led to 100% coverage for maternal health care services in Gabon today.[17] Additionally, the program has been able to cover 85% of the health costs.[17] Despite these efforts and Gabon being classified as an upper-middle income country, its health outcomes do not align with the average outcomes of an upper-middle income country. The life expectancy of Gabon is 68.51 years, the infant mortality rate 26.7 per 1000 live births and under-five year child mortality 36.3 per 1000 live births, which is respectively 76.3, 11 and 13 globally among the upper-middle income countries.[18,19] This emphasizes the necessity for further investments to improve these health outcomes.

The Gabonese Ministry of Health has currently four areas of focus covering prevention and health security, delivery of and access to health services, HIV/AIDS control and policy support.[20] Despite these intentions, the health expenditure budget remains very limited, corresponding to 1.59% of the gross national income (GNI) compared to 4.19% in other upper-middle income countries, thereby forcing them to focus on only one of the four themes of health care.[16]

1.2.3.: PRIMARY HEALTH CARE - MOZAMBIQUE

The health care system in Mozambique is divided in four levels comprising of the primary level including urban and rural health centers, the secondary level covering general, rural and district hospitals, the tertiary level covering provincial hospitals and the quaternary level including central and specialized hospitals.[21] The health centers form the first contact between patient and the health system, thereby providing essential curative services as well as vaccination and prevention of local infectious diseases.[22]

Strong commitment to the Mozambiquan PHC system has been shown by the Ministry of Health of Mozambique. In 2014, a five-year program, called the Health Sector Strategic Plan I (HSSP-I), was implemented covering multiple objectives including an increase in access and quality of health services, a reduction of geographic disparities, an increase in efficiency, strengthening sector partnerships and an increase in transparency and accountability of the Mozambican health system.[23]

© 2026 Barreix G et al. *JAMA Network Open.*

The commitment continued in 2019, in which Mozambique started strengthening the community health worker network by the PHC Care Strengthening Program. The number of trained workers increased from 1000 in 2018 to 8300 in 2022, thereby expanding community access to health services four-fold.[24] This program belonged to the Health Sector Strategic Plan II, that continued after the HSSP-I and strives to achieve UHC by 2030.[25] The objectives of this plan include an emphasis on strengthening equity in access, strengthening the distribution and education of health workforce, addressing non-communicable diseases, enhancing health financing and its sustainability and lastly focusing on digital innovations.[25]

eAppendix 1.3. Literature Search

A literature search was performed in PubMed using the Mesh Terms "Primary Health Care" AND "Developing countries" OR "Africa South of the Sahara" to analyze the existence of an effective PHC index score in previous literature. The last search was conducted on the 11th of February 2025 with limitations applied based on the type of report and year of publication of which only papers from 2000 onwards were included in the review. This search led to 2248 papers, which were screened based on their way of measuring PHC coverage. More articles were retrieved by snowballing.

To measure the functioning of the PHC system, some studies used the Primary Healthcare Performance Initiative (PHCPI) conceptual framework.[5] This framework visualizes essential elements to obtain UHC, which is commonly used as a proxy for PHC. The UHC index score is a score ranging from 0-100 covering 14 indicators divided over 4 categories, in which the indicators of the first category related to the reproductive, maternal, newborn and child health (RMNCH) form the RMNCH index score. The expanded version of this score is the Composite Coverage Index, which covers 8 indicators related to this category and thereby forms the most commonly used score in literature as a proxy for PHC.[5,25,26] No studies mention the existence of an internationally recognized effective PHC index score.

© 2026 Barreix G et al. *JAMA Network Open.*

PART II – GENERAL METHODOLOGY

eAppendix 2.1. Transformation to a Longitudinal Dataset

Step 1: Duplicate each row

First create a unique code for each individual child. In the Demographic and Health Surveys (DHS) surveys, "caseid" is used for all the children from the same mother. Combining this variable with the birth order number and survey year/phase, creates a unique code for each individual child. Sequentially, each individual code with the corresponding variables of interest is replicated five times.

Step 2: Generate 5 years backward

The last replication created in step 1 corresponds to the survey year, which is called "v007" in the DHS surveys. Starting from the survey year, we go back five years, assigning each preceding year to the preceding replication. It is important to distinguish between different calendars, such as the Ethiopian calendar versus the Gregorian calendar.

Step 3: Define and assign vital status

First, create a variable covering the month and year of birth that is given by DHS in a Century Month Code (CMC). Second, create a variable covering the month and year of death, if applicable, based on the calculated month and year of birth added by the age in months at death. The vital status per replication is then evaluated. If the child is alive during the corresponding year and did not die during that same year, it is assigned alive. If the child dies during the corresponding year, it is considered as the year of death. The follow-up stops after the year the child died or when the survey year has been reached.

eAppendix 2.2. Selection Procedure of the PHC Variables

The selection of these five variables constituting the PHC-index was based on inputs from both international and local PHC experts, literature review, and data availability. PHC can be offered during different phases of life, beginning with the preconception phase, followed by pregnancy, childbirth, the first month of life and then the subsequent four years of life. The first month of life is treated separately from the remaining years of life due to the significantly higher mortality rate during the neonatal period compared to later stages of life.[27] To represent this chronological order, FP was chosen to represent the preconceptual period, ANC was chosen to represent PHC during the pregnancy, SBA was chosen to represent medical care during birth, PNC was chosen to represent the health check-ups within the first two months of life and the perceived barriers related to distance and financial constraints were chosen to assess health care accessibility when seeking

care during the following years of life. PB was represented with the DHS variable referred to the women who reported that they have serious problems in accessing health care for themselves when they are sick.

The responses regarding FP and PB pertain to the mother, and therefore, the answers apply to all siblings within the same household. For ANC and PNC, the responses were provided only for the most recent sibling. For SBA, answers were given for children born in the last two years. If there were missing values for ANC, PNC or SBA, data from the most recent sibling was used to fill in the missing information for other siblings. The assumption hereby is that the coverage by ANC, PNC and SBA for the most recent sibling is the same for other siblings in the household with missing values for these three variables. This finally led to the five variables representing the continuity of the integrated PHC system representing the availability and accessibility of PHC services in SSA.

Other variables were also analyzed and considered for inclusion in the index, including immunizations, Oral Rehydration Therapy, child growth monitoring, nutritional supplementation and counseling, breastfeeding promotion, use of insecticide-treated nets, and treatment of fever and other symptoms, among others. However, these were not included in the final indicator because they did not meet theoretical criteria necessary to serve as suitable proxy components for integrated PHC effective coverage, or due to data availability constraints. Regarding the first issue, and given that the set of chosen variables already represented a robust proxy of effective PHC coverage, we can list: proxies of services already better represented by other included variables -such as ANC and PNC, services not universally available across countries, services limited to children with specific and narrow health conditions, or services considered weak indicators of PHC.[2,28] As for data availability, exclusion was due to factors such as lack of data on deceased children, the presence of data only for small subsamples of children, or the complete absence of data in multiple DHS surveys or countries. For example, immunization coverage was not included given that – in the countries under study and in the majority of SSA - it is an intervention more linked to vertical campaign interventions, often funded by external donors such as the Global Alliance for Vaccines and Immunization (GAVI) and others, rather than part of the an integrated PHC system. Moreover, in the dataset there was no information about the immunization status of deceased children.

Dimensions	Data on death children	Small subsample	No data on some surveys	Variable in DHS
**Immunizations**	not available			h2, h7, h9

© 2026 Barreix G et al. *JAMA Network Open.*

**Oral Rehydration Therapy**	not available	small subsample		h13
**Knowledge of ORS packages**			No observations for MZ-2022	v416
**Care seeking due to symptoms of Acute Respiratory Infection**	not available	small subsample		h32z
**Growth monitoring**			No observations for ET-2011, ET-2016, GB-2019, GB-2012	h70a, h70b, h70c
**Children who were given in the last 12 months given iron tablets, sprinkles or syrup**	not available			h42
**Medicines for intestinal worms in last 6 months**	not available			h43
**Children who were given vitamin A supplements**	not available			h33, h34
**Children who were given multiple micronutrient powders**	not available			h80a
**Breastfeeding promotion**			No observations for ET-2011, GB-2012,	m78d, m78e
**Counseled in the last 6 months about how or what to feed their child**		small subsample	No observations for GB-2019, GB-2012	v486, s413d (2011)
**Percentage of children who received Plumpy'Nut or who received Plumpy'Doz**		small subsample	No observations for GB-2019, MZ-2022	h80b, h80c
**Insecticide treated nets**			No observations for ET-2016, GB-2019	v459 (2011), m80
**During pregnancy took anti-malarial drugs**			No observations for ET-2019, ET-2016	m49a-m49y

eTable 1. DHS Variables Analyzed for Composing the PHC-Index

eAppendix 2.3. Categorization of the Covariates

The list of covariates including their categorization are presented in eTable 2 below. The categorization of "birth order", "maternal age at first birth" and "maternal education level" is based on categorizations used in literature.[29–31] The maternal education level is dichotomized based on having a low education status (no education or primary education) and a high education status (secondary or higher education). The variables "improved form of sanitation" and "improved source of drinking water" are dichotomized based on the WHO/UNICEF joint monitoring programme and the definitions used by the DHS program.[32]

© 2026 Barreix G et al. *JAMA Network Open.*

The country level data of the variables "GDP per capita PPP", "government Gross Domestic Product (GDP) spending on health" and "Gini coefficient" were retrieved from the World Bank database[19]. The country level data of the variable "stunting" was obtained via DHS STATcompiler[32]. Some values of the country level covariates were estimated through linear interpolation or linear extrapolation. Further information regarding this procedure is explained in eAppendix 2.4.

Covariates	DHS code	Label
**Demographic covariates**		
Sex of the child	b4	0 = female, 1 = male
Child's age in years	age	0, 1, 2, 3, 4, 5
**Obstetric covariates**		
Child is part of a twin	b0	0 = single birth, 1 = twin/triplets/quadruplets
Birth order	Bord	0 = 1st child<br>1 = 2nd or 3rd child<br>2 = 4th or above child
Maternal age at first birth	v212	1 = < 18 year<br>2 = 18 or older
**Socioeconomic covariates**		
Maternal education	v106	0 = no education or primary education<br>1 = secondary or higher education
Improved form of sanitation and/or drinking wáter	wash	0 = unimproved sanitation and water, 1 = improved sanitation and or improved water source
**Country-level covariates**		
GDP per capita PPP[12]	GDPpercapitaPPP	Numeric
Government GDP spending on health[12]	GDPonhealth	Percentage
Gini coefficient[12]	Ginicoef	0 to 100, 0 = equality, 100 = inequality
Stunting[12]	stunting	Percentage

eTable 2. Representing the Covariates Used in the Data Analysis, Including Their DHS Coding and the Labels Attached to Them. DHS = Demographic and Health Surveys, GDP = gross domestic product. PPP = purchasing power parity.

Categorization of sanitation and drinking water

In eTables 3 and 4, the green ones represent improved sanitation/drinking water and these are coded as "1". The white ones represent unimproved sanitation/drinking water and these are coded as "0". The orange ones represent responses from people who do not belong to that household/region or missing responses, these are coded as "missing".

© 2026 Barreix G et al. *JAMA Network Open.*

Variable water (v113)	Original coding
Piped into dwelling	11
Piped to yard/plot	12
Piped to neighbor	13
Public tap/standpipe	14
Piped to buy	15
Piped to elsewhere	16
Tube well or borehole	21
Protected well	31
Unprotected well	32
33; no label (GB 2012)	33
34; no label (GB 2012)	34
Protected spring	41
Unprotected spring	42
River/dam/lake/ponds/stream/canal/irring	43
Rainwater	51
Tanker truck	61
Cart with small tank	62
Bottled water	71
Sachet water	82
Other	96
Not a dejure resident	97
99	99

eTable 3. Green Represents Improved Drinking Water, White Represents Unimproved Drinking Water and Orange Represents Missing. "Not a dejure resident" is categorized as missing since these respondents do not usually live in that corresponding household. 33 & 34 originate from the dataset of Gabon 2012, however, no label is attached to them, therefore assuming these answers as unimproved drinking water. GB = Gabon.

Variable sanitation (v116)	Original coding
Flush to piped sewer system	11
Flush to septic tank	12
Flush to pit latrine	13
Flush to somewhere else	14
Flush, don't know where	15
Ventilated improved pit latrine	21
Pit latrine with slab	22
Pit latrine without slab/open pit	23
no facility	30
no facility/bush/field	31
composting toilet	41
Bucket toilet	42
Hanging toilet/latrine	43
No flush to piped sewer system (MZ 2022)	51
No flush to septic tank (MZ 2022)	52
No flush to somewhere else (MZ 2022)	53
No flush, don't know where (MZ 2022)	54
Other	96
Not a dejure resident	97
99	99

eTable 4. Green Represents Improved Sanitation, White Represents Unimproved Sanitation and Orange Represents Missing. "Not a dejure resident" is categorized as missing since these respondents do not usually live in that corresponding household. 51 to 54 originate from the dataset of Mozambique 2022, in which the labels indicate an unimproved form of sanitation. MZ = Mozambique.

© 2026 Barreix G et al. *JAMA Network Open.*

eAppendix 2.4. Interpolation and Extrapolation Method

In this study, "ipolate" is used to perform linear interpolation, in which the missing y values are predicted based on the association between the original x and y values that were available. The values after the last original data point are predicted by using "predict" thereby following the linear relationship of the original data points. The predicted values by linear interpolation and extrapolation should be checked by creating a new plot to evaluate the new data points in the original relationship. The results for this study are shown in eTables 5-7.[1]

Country level data based on linear interpolation/extrapolation

This procedure led to the following country level data for Mozambique:

Mozambique	GDP per capita (PPP)	Government GDP spending on health (%)	Gini coefficient	Stunting (%)
1992	**511**	0,03	49,75	46,90
1993	**549**	0,32	49,77	46,61
1994	**546**	0,62	49,79	46,32
1995	**525**	0,91	49,80	46,03
1996	**563**	1,20	**53,30**	45,74
1997	**612**	1,50	52,25	**42,40**
1998	**661**	1,79	51,20	43,17
1999	**722**	2,08	50,15	43,93
2000	**712**	**2,19**	49,10	44,70
2001	**786**	**2,88**	48,05	45,47
2002	**843**	**3,28**	**47,00**	46,23
2003	**883**	**3,4**	46,75	**47,00**
2004	**934**	**3,67**	46,50	46,45
2005	**970**	**4,38**	46,25	45,90
2006	**1042**	**4,09**	46,00	45,35
2007	**1095**	**3,93**	45,75	44,80
2008	**1142**	**4,22**	**45,50**	44,25
2009	**1179**	**5,2**	46,92	43,70
2010	**1224**	**5,44**	48,33	43,15
2011	**1276**	**5**	49,75	**42,60**
2012	**1339**	**5,25**	51,17	42,06
2013	**1386**	**6,13**	52,58	41,53
2014	**1450**	**6,33**	**54,00**	40,99
2015	**1513**	**6,72**	53,26	40,45
2016	**1538**	**7,28**	52,52	39,92
2017	**1532**	**7,84**	51,78	39,38
2018	**1539**	**8,09**	51,04	38,85

[1] Exceptionally, Health Expenditure as % of GDP for Ethiopia and Mozambique was kept unchanged until 2040 since year 2022 (replicating the 2021 values). This was assumed as continuing the linear extrapolation gave not realistic results

2019	**1529**	**7,95**	**50,30**	38,31
2020	**1466**	**7,57**	50,23	37,77
2021	**1457**	**9,05**	50,24	37,24
2022	**1477**	9,05	50,26	36,70
2023	1512	9,05	50,28	37,89
2024	1723	9,05	50,30	**37,60**
2025	1762	9,05	50,31	37,31
2026	1801	9,05	50,33	37,01
2027	1840	9,05	50,35	36,72
2028	1879	9,05	50,36	36,43
2029	1918	9,05	50,38	36,14
2030	1957	9,05	50,40	35,85

eTable 5. Representing the Country Level Data in Mozambique. Bolted numbers represent original data obtained from World Bank or DHS STATcompiler. The orange-coloured numbers represent data obtained through linear interpolation or linear extrapolation. GDP = gross domestic product. PPP = purchasing power parity.

This procedure led to the following country level data for Ethiopia:

Ethiopia	GDP per capita (PPP)	Government GDP spending on health (%)	Gini coefficient	Stunting (%)
1992	**681**	5,53	37,93	65,91
1993	**744**	5,46	37,66	64,79
1994	**741**	5,39	37,39	63,68
1995	**760**	5,32	**44,60**	62,56
1996	**827**	5,24	40,95	61,45
1997	**826**	5,17	37,30	60,33
1998	**773**	5,10	33,65	59,22
1999	**788**	5,03	**30,00**	58,10
2000	**810**	**4,37**	29,96	**57,70**
2001	**851**	**4,72**	29,92	56,32
2002	**838**	**4,71**	29,88	54,94
2003	**796**	**4,9**	29,84	53,56
2004	**877**	**4,31**	**29,80**	52,18
2005	**952**	**4,1**	30,37	**50,80**
2006	**1025**	**4,46**	30,93	49,73
2007	**1109**	**5**	31,50	48,67
2008	**1194**	**4,28**	32,07	47,60
2009	**1262**	**4,65**	32,63	46,53
2010	**1381**	**5,47**	**33,20**	45,47
2011	**1493**	**4,47**	33,56	**44,40**
2012	**1577**	**4,54**	33,92	43,20
2013	**1697**	**4,08**	34,28	42,00
2014	**1821**	**4,03**	34,64	40,80
2015	**1957**	**3,82**	**35,00**	39,60
2016	**2085**	**3,6**	31,43	**38,40**
2017	**2223**	**3,45**	31,16	37,87
2018	**2311**	**3,3**	30,89	37,33

2019	**2438**	**3,23**	30,62	**36,80**
2020	**2517**	**3,48**	30,35	34,70
2021	**2588**	**3,21**	30,08	33,58
2022	**2656**	**3,21**	29,81	32,47
2023	**2755**	3,21	29,54	31,35
2024	2581	3,21	29,26	30,24
2025	2652	3,21	28,99	29,12
2026	2722	3,21	28,72	28,01
2027	2793	3,21	28,45	26,90
2028	2864	3,21	28,18	25,78
2029	2934	3,21	27,91	24,67
2030	3005	3,21	27,64	23,55

eTable 6. Representing the Country Level Data in Ethiopia. Bolted numbers represent original data obtained from World Bank or DHS STATcompiler. The orange-colored numbers represent data obtained through linear interpolation or linear extrapolation. GDP = gross domestic product. PPP = purchasing power parity

This procedure led to the following country level data for Gabon:

Gabon	GDP per capita (PPP)	Government GDP spending on health (%)	Gini coefficient	Stunting (%)
1992	**23269**	3,36	46,75	29,30
1993	**23552**	3,33	46,40	28,72
1994	**23794**	3,31	46,05	28,14
1995	**24344**	3,29	45,70	27,56
1996	**24596**	3,27	45,35	26,98
1997	**25360**	3,25	45,00	26,40
1998	**25586**	3,23	44,65	25,82
1999	**22714**	3,21	44,30	25,24
2000	**21717**	**2,9**	43,95	**25,10**
2001	**21605**	**3,15**	43,60	24,38
2002	**20981**	**3,32**	43,25	23,67
2003	**20875**	**3,53**	42,90	22,95
2004	**20439**	**3,33**	42,55	22,23
2005	**20385**	**2,93**	**42,20**	21,52
2006	**19224**	**3,14**	41,85	20,80
2007	**19752**	**3,16**	41,50	20,08
2008	**18493**	**2,79**	41,15	19,37
2009	**17911**	**3,84**	40,80	18,65
2010	**18531**	**2,48**	40,45	17,93
2011	**19153**	**2,56**	40,10	17,22
2012	**19450**	**2,42**	39,75	**16,50**
2013	**19836**	**2,84**	39,40	16,20
2014	**20009**	**2,53**	39,05	15,90
2015	**20148**	**2,66**	38,70	15,60
2016	**19988**	**3,11**	38,35	15,30
2017	**19556**	**2,78**	**38,00**	15,00
2018	**19226**	**2,75**	37,65	14,70
2019	**19491**	**2,77**	37,30	**14,40**
2020	**18681**	**3,41**	36,95	13,06

© 2026 Barreix G et al. *JAMA Network Open.*

2021	**18524**	**2,71**	36,60	12,48
2022	**18662**	2,71	36,25	11,90
2023	**18703**	2,69	35,90	11,32
2024	17539	2,67	35,55	10,74
2025	17343	2,65	35,20	10,16
2026	17148	2,63	34,85	9,58
2027	16952	2,61	34,50	9,00
2028	16756	2,58	34,15	8,42
2029	16561	2,56	33,80	7,84
2030	16365	2,54	33,45	7,26

eTable 7. Representing the Country Level Data in Gabon. Bolted numbers represent original data obtained from World Bank or DHS STATcompiler. The orange-colored numbers represent data obtained through linear interpolation or linear extrapolation. GDP = gross domestic product. PPP = purchasing power parity

eAppendix 2.5. Different Weights Analyzed

There are 7 different weights tested to verify which weight is most appropriate. The first three weights are based on the survey design weight given by DHS in the form of the variable "v005". This survey design weight is also one of the weights that has been tested separately. The results are shown in eAppendix 3.3.1.

Option 1: Stepwise approach for equalizing the contribution of each survey

1. Take the sum of the weights (v005) per survey based on the sample you are using
2. Divide the weight (*1000000) by the sum of the weight per survey. This will equalize the contribution of each survey.

Option 2: Stepwise approach for equalizing the contribution of each country

1. Take the sum of the weights (v005) per country based on the sample you are using
2. Divide the weight (*1000000) by the sum of the weight per country. This will equalize the contribution of each country in the sample.

Option 3: Stepwise approach for equalizing the contribution of each survey within the country followed by an equal contribution of each country

1. Take the sum of the weight (v005) per survey for each country separately
2. Divide the weight (*1000000) by the sum of the weight per survey per separate country. This will equalize the contribution of each survey within each separate country.
3. Fuse the calculated weights in step 2 together in one variable. This command creates a variable in which the calculated weights that equalize the contribution of each survey within each separate country are structured in one variable.
4. Take the sum of the weights calculated in step 2 and fused in step 3. After equalizing the contribution of surveys within one country, we now want to equalize the contribution of each of the countries.

© 2026 Barreix G et al. *JAMA Network Open.*

5. Divide the weight (*1000000) by the sum of the weight per country. This will finally equalize the contribution of each of the countries.

The following three weights follow the same structure as the previous ones, however, in these cases the survey design weight given by DHS is changed.

Option 4: Stepwise approach for equalizing the contribution of each survey

1. Divide the weight of each individual child over the number of follow-up years. In this way, each original survey design weight (v005) is divided over the number of follow-up years, which means that weights of the separate observations of each individual child adds up to the same original survey design weight (v005).
2. Take the sum of the created weights in step 1 per survey based on the sample you are using
3. Divide the weight (*1000000) by the sum of the weight per survey. This will equalize the contribution of each survey.

Option 5: Stepwise approach for equalizing the contribution of each country

1. Divide the weight of each individual child over the number of follow-up years. In this way, each original survey design weight (v005) is divided over the number of follow-up years, which means that weights of the separate observations of each individual child adds up to the same original survey design weight (v005).
2. Take the sum of the created weights in step 1 per country based on the sample you are using
3. Divide the weight (*1000000) by the sum of the weight per country. This will equalize the contribution of each country.

Option 6: Stepwise approach for equalizing the contribution of each survey within the country followed by an equal contribution of each country

1. Divide the weight of each individual child over the number of follow-up years. In this way, each original survey design weight (v005) is divided over the number of follow-up years, which means that weights of the separate observations of each individual child adds up to the same original survey design weight (v005).
2. Take the sum of the created weight in step 1 per survey for each country separately
3. Divide the weight (*1000000) by the sum of the weight per survey per separate country. This will equalize the contribution of each survey within each separate country.
4. Fuse the calculated weights in step 3 together in one variable. This command creates a variable in which the calculated weights that equalize the contribution of each survey within each separate country are structured in one variable.

© 2026 Barreix G et al. *JAMA Network Open.*

5. Take the sum of the weights calculated in step 3 and fused in step 4. After equalizing the contribution of surveys within one country, we now want to equalize the contribution of each of the countries.
6. Divide the weight (*1000000) by the sum of the weight per country. This will finally equalize the contribution of each of the countries.

The final weight used in our study is option 3, based on the original survey design weight (v005), in which the contribution of each survey within the country is equalized, followed by the equalization of the contribution of each country within the sample.

eAppendix 2.6. Equation

The association of effective PHC coverage and child mortality in SSA was assessed using a two-ways fixed effects multivariable Poisson regression models[33] with child clustered standard errors. The models were weighted and adjusted for the confounding variables as well as for fixed effects by region within these three countries, and by year. The weight applied in the model was based on the survey design weight, standardized across surveys within each country, and then equalized across countries to ensure equal contribution of each country.

The equation of the model is:

$$log(VT_{irct}) = \beta_0 + \Sigma\, \beta_{\mathrm{PHC}}\, PHC_i + \Sigma\, \beta_{\mathrm{CV}}\, CV_{it} + \Sigma\, \beta_{\mathrm{AV}}\, AV_{ct} + \Sigma\, \beta_{\mathrm{R}}\, R_r + \Sigma\, \beta_{\mathrm{T}}\, T_t \quad \text{(Eq.1)}$$

Being i= child, r= region, c=country, t= year, VT= vital status, PHC= vector of PHC-index categories, CV= vector of individual covariates; AV= vector of aggregate variables, R = vector of region fixed effects, T = vector of year fixed effects, each coefficient represents the effect associated with a specific covariate, with β_0 denoting the intercept term.

PART III – DESCRIPTIVES AND RETROSPECTIVE ANALYSIS

eAppendix 3.1. Descriptives

	**Variable**	**n (%)**
**Child mortality outcome**	Child mortality < 5 year	2,166 (1.82%)
	Child mortality < 1 year	1,471 (67.87%)
	Child mortality < 1 month	1,036 (70.43%)
	Child mortality = 1 year	418 (19.30%)
	Child mortality = 2 years	187 (8.63%)
	Child mortality = 3 years	59 (2.72%)
	Child mortality = 4 years	31 (1.43%)
**PHC-index composing variables**	Family planning	26,560 (22.34%)
	Among deceased children	353 (16.30%)
	Among alive children	26,207 (22.45%)
	Antenatal care	48,864 (41.09%)
	Among deceased children	709 (32.73%)

© 2026 Barreix G et al. *JAMA Network Open.*

	Among alive children	48,155 (41.25%)
	Skilled birth attendance	52,795 (44.40%)
	Among deceased children	762 (35.18%)
	Among alive children	52,033 (44.57%)
	Postnatal neonatal care	28,499 (23.97%)
	Among deceased children	318 (14.68%)
	Among alive children	28,181 (24.14%)
	No perceived barriers in distance and/or money	29,044 (24.42%)
	Among deceased children	482 (22.25%)
	Among alive children	28,562 (24.47%)
**Effective PHC-index score**	No service	36,308 (30.53%)
	Among deceased children	872 (40.26%)
	Among alive children	35,436 (30.35%)
	Low	25,765 (21.67%)
	Among deceased children	492 (22.71%)
	Among alive children	25,273 (21.65%)
	Low-intermediate	23,674 (19.91%)
	Among deceased children	393 (18.14%)
	Among alive children	23,281 (19.94%)
	Intermediate	21,674 (18.23%)
	Among deceased children	301 (13.90%)
	Among alive children	21,373 (18.31%)
	High-intermediate	9,823 (8.26%)
	Among deceased children	97 (4.48%)
	Among alive children	9,726 (8.33%)
	High	1,667 (1.40%)
	Among deceased children	11 (0.51%)
	Among alive children	1,656 (1.42%)
**Covariates**	Male child	60,252 (50.67%)
	Childs' age in years	1.4 (average)
	Child is part of a twin	3,444 (2.90%)
	Birth order (1st or 2nd/3rd child)	40,532 (34.09%)
	Birth order (1st versus 4th or above child)	53,407 (44.91%)
	Maternal age at first birth > 18 years	68,659 (57.74%)
	Maternal education (no/primary education versus secondary or higher)	28,077 (23.61%)
	Improved form of sanitation and/or drinking water	77,616 (65,27%)
	GDP per capita in PPP (average)	6,453.79
	Percentage of governmental GDP spending on health (average)	4.64%
	Gini coefficient (average)	37.87
	National percentage of stunting (average)	34.60%
**Surveys and person-years**	Number of person-years	118,911
	Number of surveys	5

eTable 8A. Descriptive Statistics of the Study Variables. *PHC = primary health care. GDP = gross domestic product. PPP = purchasing power parity.*

	Variable		2007-2010	2011-2014	2015-2018	2019-2022
**Child mortality outcome**	Child mortality < 5 year	n	794	625	348	399
		%	2.43	1.85	1.4	1.45
**PHC-index composing variables**	Family planning	n	6,071	7,362	6,092	7,035
		%	18.56	21.75	24.56	25.55
	Antenatal care	n	9,373	12,742	10,631	16,118
		%	28.65	37.64	42.85	58.54

© 2026 Barreix G et al. *JAMA Network Open.*

	Skilled birth attendance	n	8,314	12,438	11,445	20,598
		%	25.41	36.74	46.13	74.82
	Postnatal neonatal care	n	5,909	6,936	5,279	10,375
		%	18.06	20.49	21.28	37.68
	No perceived barriers in distance and/or money	n	4,220	7,174	7,209	10,441
		%	12.9	21.19	29.06	37.92
**Effective PHC-index score**	PHC-index No service	n	15557	11961	6653	2137
		%	47.55	35.33	26.82	7.76
	PHC-index Low	n	7390	7840	5892	4643
		%	22.59	23.16	23.75	16.86
	PHC-index Low-intermediate	n	4623	6279	5028	7744
		%	14.13	18.55	20.27	28.13
	PHC-index Intermediate	n	3564	5191	4617	8302
		%	10.89	15.33	18.61	30.16
	PHC-index High-intermediate	n	1346	2244	2238	3995
		%	4.11	6.63	9.02	14.51
	PHC-index High	n	235	341	381	710
		%	0.72	1.01	1.54	2.58
**Covariates**	Male child	n	16,597	17,321	12,713	13,621
		%	50.73	51.16	51.24	49.48
	Childs' age in years	average	1.1	1.5	1.6	1.5
	Child is part of a twin	n	958	910	657	919
		%	2.93	2.69	2.65	3.34
	Birth order (1st or 2nd/3rd child)	n	10,479	11,132	8,403	10,518
		%	32.03	32.88	33.87	38.2
	Birth order (1st versus 4th or above child)	n	16,031	16,021	11,184	10,171
		%	49	47.32	45.08	36.94
	Maternal age at first birth > 18 years	n	18,859	19,759	15,228	14,813
		%	57.65	58.36	61.38	53.8
	Maternal education (no/primary education versus secondary or higher)	n	5,383	7,033	5,758	9,903
		%	16.45	20.77	23.21	35.97
	Improved form of sanitation	n	5,994	7,113	6,499	8,925

© 2026 Barreix G et al. *JAMA Network Open.*

		%	18.32	21.01	26.2	32.42
	Improved source of drinking water	n	18,431	21,308	16,333	18,062
		%	56.34	62.94	65.83	65.61
**Observations**	Person-years	n	32,715	33,856	24,809	27,531

eTable 8B. Descriptive Statistics of the Study Variables Across Time. Aggregate variables appear in eAppendix 2.4.

eAppendix 3.2. Stratification Analyses

**Model**	**IRR [95% CI]** **Female** ***(n = 58659)***	**Male** ***(n = 60252)***
PHC (low)	0.8673 [0.663-1.134]	0.7935* [0.622-1.012]
PHC (low-intermediate)	0.9809 [0.708-1.360]	0.7258** [0.547-0.963]
PHC (intermediate)	0.9305 [0.638-1.358]	0.6642** [0.476-0.927]
PHC (high-intermediate)	0.6655 [0.383-1.155]	0.6181** [0.402-0.950]
PHC (high)	0.2570** [0.073-0.904]	0.4805 [0.189-1.225]

eTable 9. Poisson Regression Models Stratified by Sex Including the Categories Female and Male. PHC = primary health care. IRR = incidence rate ratio. 95% CI = 95% confidence interval. * < 0.1, ** < 0.05, *** = 0.000.

**Model**	**IRR [95% CI]** **Age < 1** ***(n = 37583)***	**Age 1-4** ***(n = 81328)***
PHC (low)	0.8231* [0.660-1.027]	0.8180 [0.597-1.121]
PHC (low-intermediate)	0.9268 [0.724-1.187]	0.6340** [0.412-0.975]
PHC (intermediate)	0.7834 [0.579-1.060]	0.7334 [0.474-1.136]
PHC (high-intermediate)	0.7090* [0.474-1.062]	0.4882** [0.263-0.907]
PHC (high)	0.2370** [0.066-0.849]	0.7790 [0.287-2.116]

eTable 10. Poisson Regression Models Stratified by Age Including the Categories, Age Below 1 Year and 1 to 4 Years. PHC = primary health care. IRR = incidence rate ratio. 95% CI = 95% confidence interval. * < 0.1, ** < 0.05, *** = 0.000.

**Model**	**IRR [95% CI]** **Rural** ***(n = 82596)***	**Urban** ***(n = 36315)***
PHC (low)	0.8754 [0.726-1.056]	0.3828** [0.206-0.711]
PHC (low-intermediate)	0.8691 [0.677-1.115]	0.3949** [0.225-0.693]
PHC (intermediate)	1.0218 [0.755-1.383]	0.3020*** [0.170-0.537]

© 2026 Barreix G et al. *JAMA Network Open.*

PHC (high-intermediate)	0.5519** [0.320-0.952]	0.2979*** [0.158-0.563]
PHC (high)	0.6066 [0.224-1.644]	0.1629** [0.057-0.468]

eTable 11. Poisson Regression Models Stratified by Area of Residence Including the Categories, Rural and Urban. PHC = primary health care. IRR = incidence rate ratio. 95% CI = 95% confidence interval. * < 0.1, ** < 0.05, *** = 0.000.

**Model**	**IRR [95% CI]** **Poorest/poorer** ***(n = 66385)***	**Middle/richer/richest** ***(n = 52526)***
PHC (low)	0.8987 [0.712-1.135]	0.7641* [0.575-1.015]
PHC (low-intermediate)	0.9904 [0.739-1.328]	0.6687** [0.479-0.933]
PHC (intermediate)	0.7285* [0.508-1.044]	0.7601 [0.526-1.097]
PHC (high-intermediate)	0.8033 [0.457-1.412]	0.5585** [0.354-0.882]
PHC (high)	1.0599 [0.307-3.655]	0.2808** [0.104-0.755]

eTable 12. Poisson Regression Models Stratified by Wealth Index Including the Categories, Poorest/Poorer and Middle/Richer/Richest. PHC = primary health care. IRR = incidence rate ratio. 95% CI = 95% confidence interval. * < 0.1, ** < 0.05, *** = 0.000.

**Model**	**IRR [95% CI]** **Ethiopia** ***(n = 64914)***	**Gabon** ***(n = 33269)***	**Mozambique** ***(n = 20728)***
PHC (low)	0.7764** [0.642-0.938]	0.6283* [0.387-1.021]	1.1134 [0.665-1.865]
PHC (low-intermediate)	0.6922** [0.519-0.924]	0.5923** [0.385-0.911]	1.1885 [0.723-1.955]
PHC (intermediate)	0.6776* [0.448-1.026]	0.4593** [0.294-0.716]	1.3023 [0.775-2.189]
PHC (high-intermediate)	0.4198* [0.166-1.060]	0.4746** [0.274-0.822]	0.8784 [0.460-1.676]
PHC (high)	3.47e-09*** [2.28e-9-5.27e-09]	0.3717** [0.132-1.046]	0.4629 [0.145-1.482]

eTable 13. Poisson Regression Models Stratified by Country Including The Categories, Ethiopia, Gabon and Mozambique. PHC = primary health care. IRR = incidence rate ratio. 95% CI = 95% confidence interval. * < 0.1, ** < 0.05, *** = 0.000.

© 2026 Barreix G et al. *JAMA Network Open.*

eAppendix 3.3. Sensitivity and Triangulation Analyses

APPENDIX 3.3.1: SENSITIVITY ANALYSES

Potential effective PHC-index scores	Child mortality IRR [95% CI]
**PHC score: 0-5**	
PHC (low)	0.8284** (0.691-0.993)
PHC (low-intermediate)	0.8333* (0.673-1.032)
PHC (intermediate)	0.7721** (0.602-0.991)
PHC (high-intermediate)	0.6387** (0.455-0.897)
PHC (high)	0.4188** (0.193-0.909)
**PHC score: 0 vs 1-5**	
PHC (0-1)	0.8149** [0.694-0.957]
**PHC score: 0-6**	Separate perceived barriers into two categories; 1. Perceived barrier due to distance, 2. Perceived barrier due to money
PHC (0-1)	0.8726 [0.720-1.057]
PHC (0-2)	0.7976** [0.645-0.986]
PHC (0-3)	0.8626 [0.679-1.095]
PHC (0-4)	0.7851 [0.595-1.036]
PHC (0-5)	0.7119* [0.498-1.018]
PHC (0-6)	0.4385** [0.202-0.953]
**PHC score: categories (1)**	Category "prenatal": FP and/or ANC and/or SBA<br>Category "postnatal": PNC<br>Category "access": No barriers
PHC (0-1)	0.8156** [0.691-0.963]
PHC (0-2)	0.8289* [0.666-1.032]
PHC (0-3)	0.6579** [0.454-0.953]
**PHC score: categories (2)**	Category "prenatal": FP and/or ANC<br>Category "neonatal": SBA and/or PNC<br>Category "access": No barriers
PHC (0-1)	0.8242** [0.691-0.983]
PHC (0-2)	0.7770** [0.632-0.955]
PHC (0-3)	0.8773 [0.667-1.153]
**PHC score: categories (3)**	Category "prenatal": FP and ANC<br>Category "neonatal": SBA and PNC<br>Category "access": No barriers
PHC (0-1)	1.0089 [0.856-1.189]
PHC (0-2)	0.8051

	[0.606-1.070]
PHC (0-3)	0.5211* [0.243-1.118]
**PHC score: chronological order**	
At least FP	0.7778** [0.658-0.919]
At least FP & ANC	0.7098** [0.558-0.902]
At least FP & ANC & SBA	0.6471** [0.479-0.874]
At least FP & ANC & SBA & PNC	0.4060*** [0.247-0.667]
FP & ANC & SBA & PNC & PB	0.4292** [0.199-0.926]

eTable 14. Different Potential Primary Health Care (PHC) Index Scores. PHC score: 0-5 is a score ranging from 0 to 5, in which the five variables are family planning (FP), antenatal care (ANC), skilled birth attendance (SBA), postnatal neonatal care (PNC) and no perceived barriers (PB) to distance and money. PHC score: 0 vs 1-5 dichotomizes the 5 variables into either being covered by 0 services or by 1 or more. PHC score: 0-6 is a score with the same variables as in PHC score: 0-5, but the no perceived barrier to money and distance is separated into two variables. PHC score: categories (1) categorize the 5 variables into 3 categories, namely "prenatal" covering FP and/or ANC and/or SBA, "postnatal" covering PNC and "access" covering no perceived barriers to money and distance. PHC score: categories (2) categorize the 5 variables into 3 different categories, namely "prenatal" covering FP and/or ANC, "neonatal" covering SBA and/or PNC and "access" covering no perceived barriers to distance and money. PHC score: categories (3) categorize the 5 variables into 3 different categories, namely "prenatal" covering FP and ANC, "neonatal" covering SBA and PNC and "access" covering no perceived barrier to distance and money. PHC score: chronological order is a score that analyses the variables based on their chronological timeline of exposure. PHC = primary health care. IRR = incidence rate ratio. 95% CI = 95% confidence interval. * < 0.1, ** < 0.05, *** = 0.000.

**Model based on v005**	**IRR [95% CI] Original weight**	**Country weight**	**Survey weight**	**Country & survey weight**
PHC (low)	0.7972** [0.674-0.943]	0.8323** [0.695-0.997]	0.8018** [0.679-0.947]	0.8284** [0.691-0.993]
PHC (low-intermediate)	0.7655** [0.624-0.940]	0.8323* [0.672-1.031]	0.7818** [0.639-0.957]	0.8333* [0.673-1.032]
PHC (intermediate)	0.7112** [0.559-0.904]	0.7764** [0.606-0.995]	0.6894** [0.541-0.879]	0.7721** [0.602-0.991]
PHC (high-intermediate)	0.5908** [0.420-0.831]	0.6469** [0.460-0.910]	0.6041** [0.426-0.857]	0.6387** [0.455-0.987]
PHC (high)	0.3891** [0.173-0.878]	0.4477** [0.203-0.989]	0.4127** [0.179-0.954]	0.4188** [0.193-0.909]

eTable 15. Poisson Regression Models Based on Different Weights, Starting With the Original Survey Design Weight, Then Adjusted by Country, Followed by Only an Adjustment by Survey and Finally by Country and Survey. Analyses included children under-five years of age (n=37583) leading to 118911 observations. PHC = primary health care. IRR = incidence rate ratio. 95% CI = 95% confidence interval. * < 0.1, ** < 0.05, *** = 0.000.

**Model**	**IRR [95% CI] Poisson *(n = 118911)***	**OR [95% CI] Logistic *(n = 118911)***
PHC (low)	0.8284** (0.691-0.993)	0.8221** [0.681-0.992]
PHC (low-intermediate)	0.8333* (0.673-1.032)	0.8275 [0.662-1.034]
PHC (intermediate)	0.7721**	0.7650**

	(0.602-0.991)	[0.590-0.991]
PHC (high-intermediate)	0.6387** (0.455-0.897)	0.6294** [0.443-0.894]
PHC (high)	0.4188** (0.193-0.909)	0.4097** [0.186-0.901]

eTable 16. Poisson Regression Model Versus Logistic Regression Model Including the Same Covariates, Fixed Effects and Adjustment by Weight. The incidence rate ratio and the exposure are removed from the logistic regression model. The sample remains the same. PHC = primary health care. IRR = incidence rate ratio. 95% CI = 95% confidence interval. OR = odds ratio. * < 0.1, ** < 0.05, *** = 0.000.

Model based on v005/follow-up years	IRR [95% CI] Adjusted original weight	Country weight	Survey weight	Country & survey weight
PHC (low)	0.8608* [0.731-1.013]	0.8872 [0.745-1.056]	0.8640* [0.735-1.016]	0.8814 [0.740-1.049]
PHC (low-intermediate)	0.8815 [0.723-1.075]	0.9243 [0.754-1.133]	0.8963 [0.737-0.946]	0.9238 [0.754-1.132]
PHC (intermediate)	0.7856** [0.621-0.993]	0.8222 [0.647-1.045]	0.7447** [0.586-0.946]	0.8169 [0.642-1.039]
PHC (high-intermediate)	0.7028** [0.501-0.987]	0.7443* [0.532-1.042]	0.7214* [0.508-1.027]	0.7302* [0.522-1.021]
PHC (high)	0.3654** [0.157-0.853]	0.4120** [0.178-0.952]	0.4017** [0.168-0.962]	0.3839** [0.168-0.876]

eTable 17. Poisson Regression Models Based on Different Weights, Starting With the Original Survey Design Weight Divided by the Number of Follow-Up Years, Then Adjusted by Country, Followed by Only an Adjustment by Survey and Finally by Country and Survey. Analyses included children under-five years of age (n=37583) leading to 118911 observations. PHC = primary health care. IRR = incidence rate ratio. 95% CI = 95% confidence interval. * < 0.1, ** < 0.05, *** =

APPENDIX 3.3.2: TRIANGULATION ANALYSES

Model	IRR [95% CI] Longitudinal dataset *(n = 118911)*	IRR [95% CI] Pooled cross-sectional dataset *(n = 37583)*
PHC (low)	0.8284** (0.691-0.993)	0.8272** [0.695-0.984]
PHC (low-intermediate)	0.8333* (0.673-1.032)	0.8218* [0.669-1.011]
PHC (intermediate)	0.7721** (0.602-0.991)	0.7567** [0.595-0.963]
PHC (high-intermediate)	0.6387** (0.455-0.897)	0.6412** [0.459-0.895]
PHC (high)	0.4188** (0.193-0.909)	0.4322** [0.197-0.947]

eTable 18. Poisson Regression Model for the Longitudinal Dataset Versus Poisson Regression Model for the Pooled Cross-Sectional Dataset. In the case of the later model, the fixed effects year is removed due to multicollinearity and the model is not adjusted by exposure, since each child has the same time period of exposure. PHC = primary health care. IRR = incidence rate ratio. 95% CI = 95% confidence interval. * < 0.1, ** < 0.05, *** = 0.000.

Model	IRR [95% CI] Longitudinal dataset *(n = 118911)*	IRR [95% CI] Pooled cross-sectional dataset *(n = 37583)*
PHC (low)	0.8221** [0.681-0.992]	0.8152** [0.676-0.984]

© 2026 Barreix G et al. *JAMA Network Open.*

PHC (low-intermediate)	0.8275* [0.662-1.034]	0.8102* [0.648-1.014]
PHC (intermediate)	0.7650** [0.590-0.991]	0.7418** [0.573-0.960]
PHC (high-intermediate)	0.6294** [0.443-0.894]	0.6227** [0.437-0.887]
PHC (high)	0.4098** [0.186-0.901]	0.4140** [0.184-0.933]

eTable 19. Logistic Regression Model for the Longitudinal Dataset Versus Logistic Regression Model for the Pooled Cross-Sectional Dataset. In the case of the later model, the fixed effects year is removed due to multicollinearity and the model is not adjusted by exposure, since each child has the same time period of exposure. PHC = primary health care. IRR = incidence rate ratio. 95% CI = 95% confidence interval. * < 0.1, ** < 0.05, *** = 0.000.

QUASI-EXPERIMENTAL TRIANGULATION

To increase the confidence in the causal inference of these impact evaluations, we performed alternative triangulation analyses, including Inverse Probability Treatment Weighting (IPTW) multivariable Poisson regressions and propensity score matching analyses (PSM).

We utilized multivariable Poisson regression models adjusted for all relevant confounding variables at both the individual and aggregate levels, with follow-up time as an offset variable, robust standard errors, and observations weighted using stabilized, truncated, IPTW.

In the eTable 20 are shown the different exposure constructions tested. For both impact evaluation methods, a binary exposure variable with two categories is needed, exposed and not exposed. Four different versions were created and assessed: PHC_1, PHC_2, PHC_3, and PHC_4.

The model we adopted and considered the most appropriate, based on avoiding contamination in the exposure category: is PHC_2. The estimates from this model are consistent and align with those from the main analysis Poisson model, PHC-4. Using PHC_2, was also conducted in the PSM analysis, which produced results of similar magnitude.

**Variables**	**Child mortality - IRR (95% CI)**								
	**Poisson**				**Poisson -IPTW**				**PSM**
	**PHC-1**	**PHC-2**	**PHC-3**	**PHC-4**	**PHC_1**	**PHC_2**	**PHC_3**	**PHC_4**	**PHC_2**
**PHC-index score**									
PHC *(low)*	**base**	**base**	**base**	**base**	**base**	**base**	**base**	**base**	**base**
PHC (*low-intermediate*)	0.7812** (0.653-0.935)	0.8320* (0.693-0.998)	0.8231* (0.688-0.985)	**0.8354 (0.697-1.001)**		**0.7769*** (0.6915-0.9728)**	**x**	**x**	**0.6955*** (0.6190-0.8151)**
PHC (*intermediate*)	0.7715** (0.641-0.929)	0.8635 (0.698-1.068)	0.8075* (0.652-0.999)	**0.8402 (0.677-1.042)**	**0.7898* (0.6851-0.9105)**		**x**	**x**	
PHC (*high-intermediate*)	0.6621*** (0.544-0.806)	0.7841 (0.607-1.012)	0.7276* (0.567-0.933)	**0.7743* (0.601-0.998)**			**x**	**x**	
PHC (*high*)	0.5194*** (0.389-0.694)	0.6180** (0.443-0.861)	0.5904** (0.425-0.820)	**0.6418* (0.455-0.906)**			**0.5021* (0.3236-0.7789)**	**x**	
PHC *(low)*	0.3151**	0.3784*	0.3845*	**0.4193* (0.192-0.918)**				**0.1298*** **	

	(0.148-0.670)	(0.175-0.820)	(0.177-0.836)					**(0.0545-0.3093)**	
				**Obs.:118,221**	**Obs.:118,221**	**Obs.:118,221**	**Obs.: 46,577**	**Obs.: 29,149**	
**Demographic covariates**									
Male child			1.2990*** (1.136-1.485)	1.2985*** (1.136-1.484)					
Child's age in years			0.2512*** (0.225-0.280)	0.2514*** (0.225-0.281)					
**Obstetric covariates**									
Child is part of a twin			4.0546*** (3.280-5.012)	4.0983*** (3.318-5.061)					
Birth order (1st or 2nd/3rd child)			0.6959*** (0.580-0.835)	0.6877*** (0.573-0.826)					
Birth order (1st versus 4th or above child)			0.7721** (0.646-0.923)	0.7480** (0.623-0.897)					
Maternal age at first birth in years >= 18			0.8591* (0.750-0.984)	0.8610* (0.752-0.986)					
**Socioeconomic covariates**									
Maternal education >= 7 years				0.8125* (0.662-0.997)					
Improved form of sanitation				0.9822 (0.806-1.196)					
Improved source of drinking water				0.9824 (0.846-1.140)					
**Country level covariates**									
GDP per capita in (PPP)				1.000 (0.100-0.100)					
Percentage of governmental GDP spending on health				0.9960 (0.768-1.291)					
Gini coefficient				1.0167 (0.842-1.228)					
National percentage of stunting				1.0636 (0.846-1.338)					
Region and Year fixed effects	No	Yes	Yes	Yes					

eTable 20. Crude Model (PHC-1) on the Left Side of the Table, Purely Covering the Impact Between the Primary Health Care Index Score and Vital Status, Followed by Adding Fixed Effects for Year and Region (PHC-2), Followed by the Addition of the Demographic and Obstetric Covariates (PHC-3) and Finalizing With the Addition of the Socioeconomic and Country Level Covariates (PHC-4). Analysis included children under-five years of age (n=37,583) leading to 118,911 observations. PHC_1, PHC_2, PHC_3 and PHC_4 is the different approached tested in the IPTW triangulation. Then, PHC_2 specification was used for the PSM triangulation. IRR = incidence rate ratio. 95% CI = 95% confidence interval. GDP = gross domestic product. PPP = purchasing power parity. * < 0.05, ** < 0.001, *** = 0.000.

© 2026 Barreix G et al. *JAMA Network Open.*

Variables	Poisson	Poisson -IPTW	PSM
	**PHC-4**	**PHC_2**	**PHC_2**
**PHC-index score**			
PHC (*no service*)	**base**	**base**	**base**
PHC *(low)*	0.8354<br>(0.697-1.001)		
PHC (*low-intermediate*)	0.8402<br>(0.677-1.042)		
PHC (*intermediate*)	0.7743*<br>(0.601-0.998)	0.7769***<br>(0.6915-0.9728)	0.6955***<br>(0.6190-0.8151)
PHC (*high-intermediate*)	0.6418*<br>(0.455-0.906)		
PHC (*high*)	0.4193*<br>(0.192-0.918)		
**Demographic covariates**			**Logistic regression**
Male child	1.2985***<br>(1.136-1.484)	1.2080***<br>(1.0918-1.3366)	1.0059<br>(0.9824-1.0299)
Child's age in years	0.2514***<br>(0.225-0.281)	0.2676***<br>(0.2474-0.2895)	0.9512***<br>(0.9449-0.9574)
**Obstetric covariates**			
Child is part of a twin	4.0983***<br>(3.318-5.061)	4.5722***<br>(3.9221-5.3300)	1.2231***<br>(1.1399-1.3125)
Birth order (1st or 2nd/3rd child)	0.6877***<br>(0.573-0.826)	0.7558***<br>(0.6578-0.8677)	0.8306***<br>(0.8010-0.8614)
Birth order (1st versus 4th or above child)	0.7480**<br>(0.623-0.897)	0.7628***<br>(0.6681-0.8710)	0.7004***<br>(0.6768-0.7248)
Maternal age at birth in years >= 18	0.8610*<br>(0.752-0.986)	0.9053<br>(0.8158-1.0046)	0.9972<br>(0.9732-1.0218)
**Socioeconomic covariates**			
Maternal education >= 7 years	0.8125*<br>(0.662-0.997)	0.7842*<br>(0.6712-0.9162)	3.9904***<br>(3.7924-4.1988)
Improved form of sanitation	0.9822<br>(0.806-1.196)	0.8644*<br>(0.7499-0.9963)	2.2143***<br>(2.1272-2.3051)
Improved source of drinking water	0.9824<br>(0.846-1.140)	0.9997<br>(0.8816-1.0970)	1.9499***<br>(1.9030-1.9980)
**Country level covariates**			
GDP per capita in (PPP)	1.000<br>(0.100-0.100)	0.9997<br>(0.9995-1.0000)	0.9997***<br>(0.9996-0.9998)
Percentage of governmental GDP spending on health	0.9960<br>(0.768-1.291)	0.9762<br>(0.7119-1.0783)	0.9130*<br>(0.8517-0.9787)
Gini coefficient	1.0167<br>(0.842-1.228)	1.0530<br>(0.9049-1.2253)	1.2152***<br>(1.1593-1.2739)
National percentage of stunting	1.0636<br>(0.846-1.338)	1.1255<br>(0.9268-1.3669)	1.0169<br>(0.9597-1.0774)
Region and Year fixed effects	Yes	Yes	Yes

eTable 21. PHC-4 for Which Analysis Included Children Under-Five Years of Age (n=37,583) Leading to 118,911 Observations; PHC_2 for the IPTW and PSM Triangulation. IRR = incidence rate ratio. 95% CI = 95% confidence interval. GDP = gross domestic product. PPP = purchasing power parity. * < 0.05, ** < 0.001, *** = 0.00

eAppendix 3.4. Bradford Hill Causality Criteria

We adopted the consolidated causal inference Bradford Hill criteria.[34] In epidemiology, causality is established according to several criteria, the oldest and most consolidated are Bradford Hill`s criteria, which include 1) Strength of the association, 2) Consistency, 3) Specificity, 4) Temporality, 5) Biological gradient, 6) Plausibility, 7) Coherence 8) Experiment 9) Analogy.

© 2026 Barreix G et al. *JAMA Network Open.*

Our analytical strategy was developed to strengthen, as much as possible, any causal inference from the statistical associations we found through each of these 9 criteria, as summarized in eTable 22.

**Bradford Hill`s criteria**		**Analytical strategy used in the paper**
**Criteria**	**Meaning**	
1. Strength of the association	The stronger the association between exposure and effect (e.g., high relative risk), the more likely it is to be causal.	The **strong effect** and statistical significance of PHC coverage over reductions in child mortality ensure compliance with Bradford Hill's first criterion.
2. Consistency	If different studies, across various populations and settings, observe the same association, it strengthens the causal hypothesis.	The protective relation of PHC on child mortality has already been study in previous studies in other country populations, including the **Latin American region and Low- and middle-income countries.**[35–38]
3. Specificity	If a specific exposure leads to a single effect (or a limited number of effects), this supports causality.	The model is carefully **adjusted for confounders** at the individual, household, and national levels. Additionally, it incorporates subregional and year fixed effects. These adjustments ensure a precise assessment of the specific impact of PHC coverage on child mortality.
4. Temporality	The exposure must precede the effect in time (a fundamental principle for establishing causality).	The use of **longitudinal panel data** ensures the evaluation of temporal relationships insure the evaluation of temporal relationships. Also, most of the variables that conform the PHC-index by definition happened before the outcome,
5. Biological gradient	(Dose-Response Relationship) – If increased exposure levels correspond to a greater risk of the effect, this supports causality.	Working with categorical variables with a natural order, enabled **dose-response** analysis between different levels of coverage (from lowest to highest) and mortality. **The results show a clear gradient** (dose-response) in relation to the effects of coverage levels on mortality.<br><br>Additionally, we estimated models using a **dichotomous coverage** definition and **IPTW** and **PSM** (in triangulations) which showed to be consistent, in direction and magnitude, to the main specification, providing a **strong support for a dose-response relationship**, in line with Bradford Hill's biological gradient criterion.
6. Plausibility	The proposed causal relationship should align with existing scientific knowledge and biological mechanisms.	The plausibility of PHC coverage effects on reducing child mortality rates is illustrated in the **WHO´s PHC framework.**[5]
7. Coherence	The relationship should be consistent with what is already known about the disease or effect being studied.	The PHC coverage effect on child mortality is coherent with all the evidence and theoretical knowledge about the positive effect of **UHC financial and physical access to health services**[35]**, or different Maternal and Child Health interventions** on child mortality.[39–44]
8. Experiment	If experiments (such as clinical trials or natural experiments) confirm the association, this strengthens the causal inference.	While this criterion establishes that randomized experiments generally offer stronger causal inference than other study designs, it also acknowledges that even randomized studies may yield weak causal conclusions depending on their design specifications. More importantly, it does not exclude the possibility of deriving causal inference from quasi-

Bradford Hill`s criteria		Analytical strategy used in the paper
**Criteria**	**Meaning**	
		experimental or observational designs. Carefully conducted, robust observational studies that adhere to causality criteria and include comprehensive sensitivity and triangulation analyses can provide a sufficient level of causal inference.<br><br>However, our combination of **IPTW with PSM** (triangulation analysis) serves as a **quasi-experimental** approach, simulating conditions of a randomized controlled trial by reducing selection bias (through matching) and balancing confounders (through weighting). This strengthens the **internal validity** of the results and supports the causal interpretation of the association between PHC coverage and child mortality reduction.
9. Analogy	If a similar association has been demonstrated between another factor and a comparable effect, it may suggest causality.	Several studies analysing the effect of **different Maternal and Child Health interventions** on child health and child mortality, showing analogous relations exist. [39–44]

eTable 22. Bradford Hill Causality Criteria

eMethods 2. Forecasting Analysis

PART I – DATA SOURCES AND GENERAL CONTEXT

eAppendix 4.1. Scenarios Contextual Background

The Institute for Health Metrics and Evaluation (IHME) provides data on the composition of health sector funding in the countries analyzed, including the Development Assistance for Health (DAH) funds.[1]

© 2026 Barreix G et al. *JAMA Network Open.*

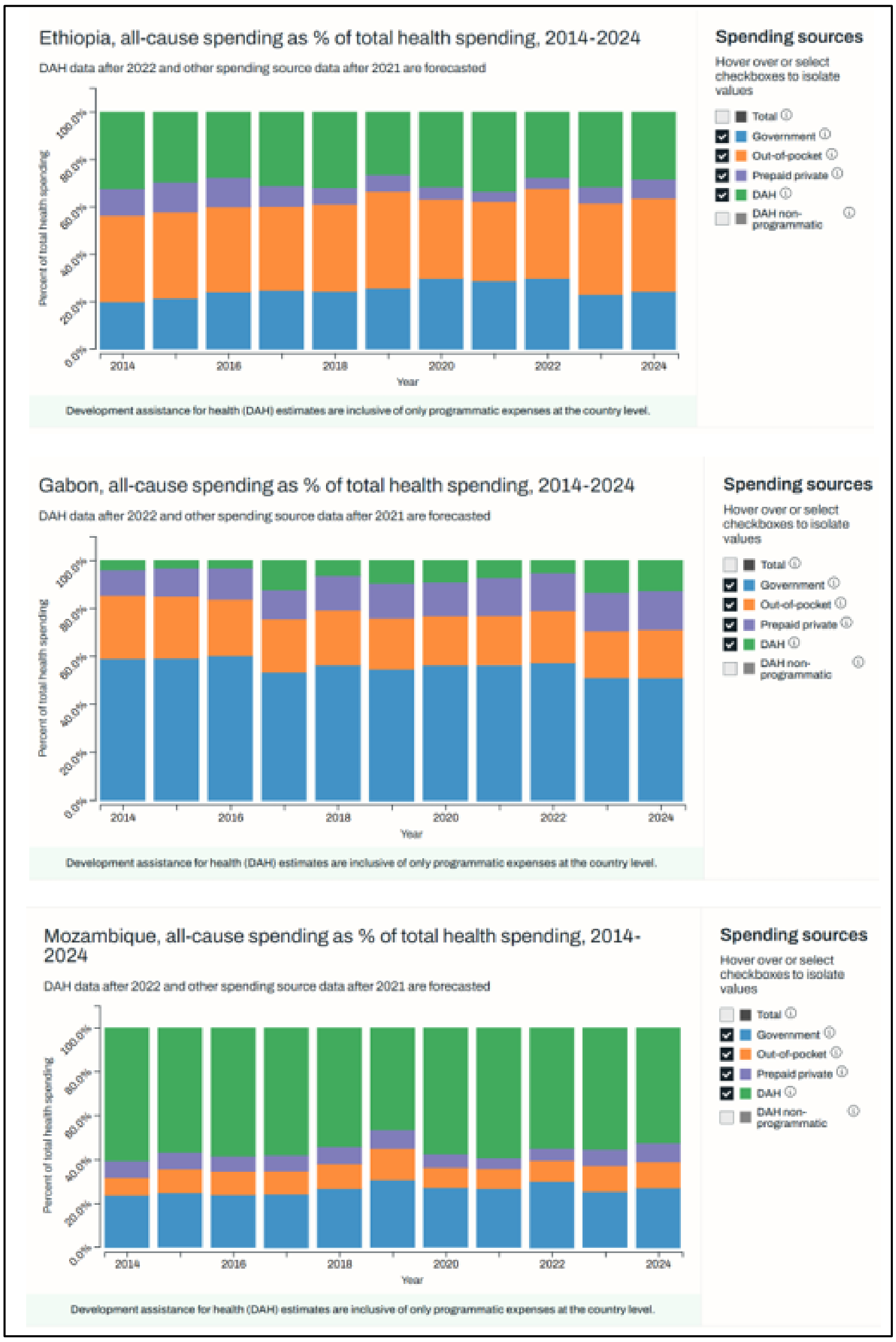

eFigure 2. Bar Graphs on Data About Spending Sources % Over Total Health Spending by Analyzed Country, Period 2014-2024 Source: Institute for Health Metrics and Evaluation[1]

DAH by definition, is provided by governments and private organizations in high-income countries to low- and middle-income countries. The provision of DAH is thus influenced by the prevailing economic and political trends in Organisation for Economic Co-operation and Development (OECD) countries. Austerity policies and budget cuts in OECD countries have

© 2026 Barreix G et al. *JAMA Network Open.*

impacted their commitments to development assistance, leading to reductions or shifts in funding priorities.

DAH % of total health spending (2014 - 2024)											
	2014	2015	2016	2017	2018	2019	2020	2021	2022	2023	2024
Ethiopia	32,19	29,54	27,77	31,15	31,76	26,36	31,65	33,43	27,50	31,35	28,49
Gabon	3,87	3,02	3,21	12,11	6,18	9,44	8,94	7,28	5,23	13,58	12,67
Mozambique	60,57	56,56	58,14	58,04	54,24	46,47	57,6	58,98	54,82	55,15	52,43

eTable 23. DAH % as of Total Health Spending by Analyzed Country, 2014-2024 Source: Institute for Health Metrics and Evaluation[1]

DAH % of total health spending (2014 - 2024)			
	MIN	MAX	MEDIA
Ethiopia	26,36	33,43	30,11
Gabon	3,02	13,58	7,78
Mozambique	46,47	60,57	55,73

eTable 24. DAH % as of Total Health Spending (Minimum, Maximum and Average) by Analyzed Country, 2014-2024 Source: Institute for Health Metrics and Evaluation[1]

The *Defunding Scenario* (see Manuscript or eAppendix 5.3) assumes that certain percentage of individuals move to PHC-index score of zero in 2025, considered the average of DAH contributions to health financing in the studied countries from 2014 to 2024 (30.1%, 7.8% and 55.7% for, Ethiopia, Gabon and Mozambique, respectively). The scenario given reflects the heavy dependency of SSA health systems financing in external sources.

According to the WHO Global Health Expenditure Database (GHED), in Ethiopia, between 2016 and 2020, more than 70% of the external expenditure was allocated to strengthen PHC services, while in Gabon the percentage is even more significant- more than 90% of external resources were implemented to boost PHC. This data highlights that PHC services are highly vulnerable to external financing sources fluctuations, being at high risk of sudden interruptions in case of aid cuts.[45]

The current scenario of the aid sector and DAH to SSA health systems support the decision made in this study to calculate *Defunding Scenario*. In the begging of 2025 United States announced the interruption of more than 80% of its projects carried out by the United States Agency for International Development (USAID), the country aid agency. USA is historically the biggest donor to humanitarian and development sectors and of health activities in SSA countries. In 2023, USA contribution to the DAH represented 31,8% of total dollars allocated by all other countries.[1,46]

The sudden cuts caused the immediate interruption of life saving activities without progressive reductions, not allowing countries to prepare funding alternatives to maintain services. After the dismantling of USAID, other western donor countries announced reduction aid budgets in the

© 2026 Barreix G et al. *JAMA Network Open.*

next 3-5 years, United Kingdom (40%), France (37%), the Netherlands (30%) and Belgium (25%).[47] There is currently a reduction trend in aid budget towards SSA countries.

A study recently released, provide a comprehensive evaluation of the impact of total USAID funding—including support for healthcare, nutrition, development, humanitarian relief, education, and other sectors—on mortality rates in low- and middle-income countries (LMICs) over the past two decades and forecasted the effects of defunding on mortality up to 2030. Forecasting models predicted that the current steep funding cuts could result in more than 14.0 million (95%Uncertainty Interval UI:8.4-19.7) additional all-age deaths, including 4.5 million (95%UI:2.7-5.9) under-five deaths, by 2030.[48]

Therefore, we can conclude that PHC services in SSA countries are highly susceptible to external funding fluctuations. The 2025 aid sector scenario brings a lot of uncertainty, and the abrupt interruptions of services could have devasting effects on child mortality, such as had been projected in this study.

eAppendix 4.2. Dataset

Data sources

We generated a synthetic cohort (SC) based on cross-sectional survey child representative data by country. The main data source for creating the SC were the DHS surveys.[32] This includes representative at national level data on under-five child in each country. We used the Mozambique Demographic and Health Survey 2022-23, the Gabon Demographic and Health Survey 2019-21 and the Ethiopia Demographic and Health Survey 2016.

All the variables used in the study came from the DHS data, except for the aggregate variable, that came from World Bank Data, and the prevalence of stunting that came from DHS (Stat compiler) data.[19,32]

Variables

The variables included (i.e., main variables and covariates) have had the same definition and treatment as expressed for the retrospective analysis, as detailed previously in this document.

Input parameters

The input parameters related to the effect of the different variables were the ones estimated by the main results of the retrospective analysis.

PART II – FORECASTING ANALYSIS

eAppendix 5.1. Description of the Forecasting Methodology

© 2026 Barreix G et al. *JAMA Network Open.*

This section details the forecasting methodology in accordance with the international modelling reporting guidelines ISPOR-SMDM.[49] In the first stage, a SC of all under-five children for the period 2011 (2012 for Gabon) until 2035 was created as a post-sample forecasting of the DHS`s surveys for each country. In the second stage, for each child and year in the sample, its mortality (i.e., death or alive state) was estimated as the outcome of a Poisson function including an intercept and a secular trend, the PHC-index data, sociodemographic characteristics, obstetric data, and country aggregate variables as input values.

eAppendix 5.2. Purpose of the Forecasting and its Applications

The model aims to simulate the effects of different PHC-index policy scenarios (as detailed in eAppendix 4.1) on child mortality in each country using individual-level and ecologic-level data as inputs.

eAppendix 5.3. First Stage: Synthetic Cohort Creation

Cohort

The general strategy for creating the SC was to include the under-five alive children sampled in the country DHS survey, as a representative sample of the under-five children population in the first year considered for the country (i.e., year 2011 for Mozambique and Ethiopia and year 2012 for Gabon). To populate the following years until 2035, the children were followed until reaching five years old (with and adjustment of some projected covariates across years). Also, we included the newly born in each of following years after the first year (explained in next paragraph). This results in a baseline first cohort year (i.e., 2011 for Mozambique and Ethiopia or 2012 for Gabon) including the under-five population and a following yearly addition of newly born in each year. Also, as a child gets to be five or more years it is out of the cohort.

To better explain the procedure, calling sub-cohort to the children entering the cohort each year within the 2011/2-2040 spam period, in operative terms, the following was applied:

1) Generate the initial year sub-cohort of children (starting in 2011/12).
    1. Replicate the DHS under-five data in each of the following years until 2040 (at the end of the steps procedure the children over five years of age were taken out of the cohort).
2) Generate the next entering sub-cohort of children by:
    1. Subsample the initial year sub cohort data (starting in 2012/13).
    2. In this last subset, adjust the children age so as they are 0 age in the first year and increase their age as years pass.

© 2026 Barreix G et al. *JAMA Network Open.*

3) Do the previous step for all entering sub-cohorts based on the previous sub-cohort.
4) Merge all the yearly sub-cohorts to constitute the SC.
5) Project the individual-level time varying variables (as explained after in this document)

The underlying assumption is that in general terms the DHS survey data used, which included representative child distribution of the variables used to create the PHC-index and the other associated data, in addition to an adjustment of some covariate across time (i.e., projected based on aggregated variables trends as explained after), would be a good proxy for representing the under-five population across the period spam.

As commented, some variables were varied across time. These variables were sanitation, water access and mother´s education. For this, the proportion of under-five children in each category of the variables were computed for the last two DHS`s surveys in each country. This proportion was linearly extrapolated until 2035. Lastly, different children were transitioned between categories stochastically to approximate the computed proportions in the first and last year of the cohort. The results can be seen in eAppendix 5.5.[2]

Scenarios

The three scenarios analysed are the *Baseline Scenario (business-as-usual BAU)*, a *Defunding Scenario* and a Universal PHC Coverage Scenario, defined as:

1. *Baseline Scenario*: the children PHC-index was held unchanged until 2035.
2. *Universal PHC Coverage Scenario*: the individuals started to transition, from 2025, to category five of the PHC-index, by increasing one category per year, until everyone is in category 5, and remained there until 2040.
3. *Defunding Scenario*: in the *Defunding Scenario* X% (55.7% Mozambique, 7.9% Gabon, 30.1% Ethiopia, see eAppendix 4.1) of individuals transitioned to the zero category in 2025 (starting with children in higher PHC-index category scores until reaching the mentioned %).

Expansion

After having created the SC for all the period of analysis, based on the DHS cross-sectional survey sample-level observations, we expanded it to account for population-level number of observations. The reasons to expand the survey are: 1- need to respect the sample weighting; 2- generate more accurate results; 3- represent children in the countries at the national level.

[2] Exceptionally, Health Expenditure as % of GDP for Ethiopia and Mozambique was kept unchanged until 2040 since year 2022 (replicating the 2021 values). This was assumed as continuing the linear extrapolation gave not realistic results.

For this, the following weights (kidwt) were computed for conducting the expansion:

kidwt = (v005/ TSO) x (POP) x EXP_%

v005 = individual survey weight

TSO = sum of v005 in the year

POP = number of individuals in the country

EXP_% = the proportion of the POP by which the expansion is intended.

Because of computational issues, the sample was expanded to the 10% for Mozambique and Ethiopia (i.e., EXP_% = 0.1). Because of this, the model estimated number of deaths are later multiplied by the inverse of EXP_% in order get total number of deaths at the population level. As we are still expanding it to sufficiently large numbers, we will not be losing representativeness.

The population aggregates used come from United Nations Department of Economic and Social Affairs, Population Division data (UNDP data)[50]:

1) For the first year (2011 for Mozambique and Ethiopia and 2012 for Gabon) we used 1-year specific age count (from 0 to 4) for each age specific group. To that, because the population counts would be already subtracting deaths, we added the age specific deaths counts (from 0 to 4).

Age	Population	Deaths
2011 Ethiopia		
0	3177314	173272
1	3114068	25347
2	3086384	22632
3	3063432	20175
4	3023769	17762
2011 Mozambique		
0	941459	73509
1	890057	10775
2	848704	6448
3	801517	4156
4	762762	2813
2012 Gabon		
0	57642	2415
1	55549	415
2	53237	243
3	50934	160
4	48902	109

eTable 25. Under-Five Population and Under-Five Deaths for 2011 Ethiopia, 2011 Mozambique and 2012 Gabon Sub-Cohorts, by Age Group

2) The sub-cohorts that are added each year are the livebirths from the Births variable.

3) For year 2024 and onwards, for the before mentioned aggregates we used the "medium variant" estimates.

eAppendix 5.4. Second Stage: Prediction Methodology

To predict the mortality of each child included in the SC, in each year, a Poisson function was used to compute the lambda of the Poisson distribution, given their set of associated variables. With that lambda, we implemented a Poisson distribution prediction to predict the mortality (if the prediction was 0, then the status is alive, and if it is 1 or greater is allocated as dead). A national level intercept and time secular trend were included to account for not explicitly modelled characteristics.

The approach for computing the lambda used the following equation:

$$Lambda_{it} = e^{(\beta 0 + Tt + \beta v\ Xit)}$$

Where i= child, t=year, β_0 = national intercept, T_t= secular trend term, β_v= vector of effect coefficients of variables, X_{it} = set of covariates. The secular trend term is $e^{(\beta 1 * (t\text{-initial year}))}$

1,000 simulations of the whole period span for each policy scenario were conducted using the Monte Carlo sampling method.

In each simulation the intercept coefficient was sampled based on a normal distribution. The standard deviation of this was set by calibration: by varying the standard deviation until approximating (of up to 0 decimals after the comma) the model´s resulting U5MR uncertainty interval width in the initial year with the UN Inter-agency Group for Child Mortality Estimation [51](IGME) U5MR confidence interval[3].

Outputs
The immediate output of the model is the mortality (i.e., death or alive) of the child in every year. Based on it, for each scenario, the resulting mean number of dead children (translated in number of preventable deaths between the *Baseline PHC Coverage Scenario* and the *Defunding Scenario*), the Rate Ratios (between *Baseline PHC Coverage Scenario* and the *Defunding Scenario* in each year) and U5MR (number of deaths per 1,000 live births) in every year obtained through the Monte Carlo simulations were computed.

[3] Given that the IGME U5MR confidence interval varied greatly across years, it was assumed to be as of the average % deviation (upper and lower) from the point estimate U5MR, in the period 2011/12-2023.

Calibration

For the calibration we used under-five child mortality estimates from IGME.[51]

The calibration had two steps:

1. We calibrated the national intercept model by approximating the IGME Mozambique 2011, Gabon 2012 and Ethiopia 2011 U5MR´s up to cero digits after comma, with the ones estimated by our model in the years 2011, 2012 and 2011, respectively.
2. We calibrated the secular trend parameter by approximating (up to 2 decimals after the coma) the exponential decay parameter resulting from fitting the model´s yearly 2011/12-2040 U5MR´s with an exponential function, and, the exponential decay parameter resulting from fitting the IGME U5MR data available (until 2023 inclusive) with an exponential function.

Validation

The validation was conducted by comparing the resulting U5MR`s of the model and UNDP data[50], for which data is available.

As it can be appreciated, almost all the UNDP estimates fall within our model estimate uncertainty intervals.

© 2026 Barreix G et al. *JAMA Network Open.*

Country	Year	Model U5M	Model lower	Model upper	UNDP U5M
Mozambique	2011	90.5	78.4	103.7	99.7
	2012	89.5	77.8	102.6	95.3
	2013	85.7	74.6	98.2	91.3
	2014	83.9	72.9	95.8	87.4
	2015	79.2	68.9	90.5	83.9
	2016	73.7	64.3	84.1	80.6
	2017	69.2	60.1	79.1	77.6
	2018	64.9	56.5	74.4	75
	2019	61.9	54.1	71.0	72.8
	2020	59.5	51.8	68.2	70.5
	2021	54.8	47.5	62.8	68.3
	2022	52.7	45.8	60.5	66.4
	2023	56.5	49.2	65.0	64.3
	2024	55.6	48.3	63.7	63
	2025	54.1	46.9	62.1	61.9
	2026	52.7	45.7	60.6	60.7
	2027	51.3	44.3	58.9	59.5
	2028	50.0	43.4	57.4	58.5
	2029	48.7	42.3	55.8	57.4
	2030	47.5	41.0	54.6	56.3
Gabon	2012	48.7	38.5	60.7	56.9
	2013	48.9	39.1	60.9	54.5
	2014	49.4	39.4	61.2	51.9
	2015	49.1	39.0	61.0	49.5
	2016	47.5	38.0	58.9	47.3
	2017	46.2	36.6	57.0	45.4
	2018	44.9	35.6	55.7	43.8
	2019	45.4	35.7	56.3	42.5
	2020	39.7	31.5	49.2	41.2
	2021	39.0	30.8	48.4	39.8
	2022	38.4	30.5	47.4	38.6
	2023	37.4	29.7	46.4	37.4
	2024	33.7	26.9	42.0	36.3
	2025	32.6	25.9	40.6	35.3
	2026	31.4	24.8	39.1	34.4
	2027	30.2	23.8	37.8	33.5
	2028	29.1	23.1	36.2	32.7
	2029	27.9	21.9	34.8	31.9
	2030	27.1	21.4	33.8	31.1
Ethiopia	2011	78.8	64.2	96.6	78.3
	2012	75.9	61.4	92.6	74.1
	2013	73.2	59.4	89.1	70.4
	2014	69.4	56.4	84.3	66.7
	2015	66.4	54.0	80.7	63.3
	2016	60.6	49.2	73.8	60.3
	2017	60.5	49.1	73.7	57.4
	2018	59.6	48.5	72.8	54.8
	2019	59.3	48.2	72.2	52.4
	2020	53.0	43.1	64.6	50.4
	2021	51.1	41.3	62.3	48.4
	2022	48.9	39.7	59.7	46.5
	2023	46.9	38.1	57.3	44.6
	2024	43.9	35.6	53.5	43.2
	2025	42.0	34.0	51.4	41.9
	2026	40.1	32.6	49.2	40.5
	2027	38.4	31.1	47.0	39.0
	2028	36.8	29.8	45.0	37.6
	2029	35.2	28.5	43.1	36.3
	2030	33.5	27.2	41.0	35.0

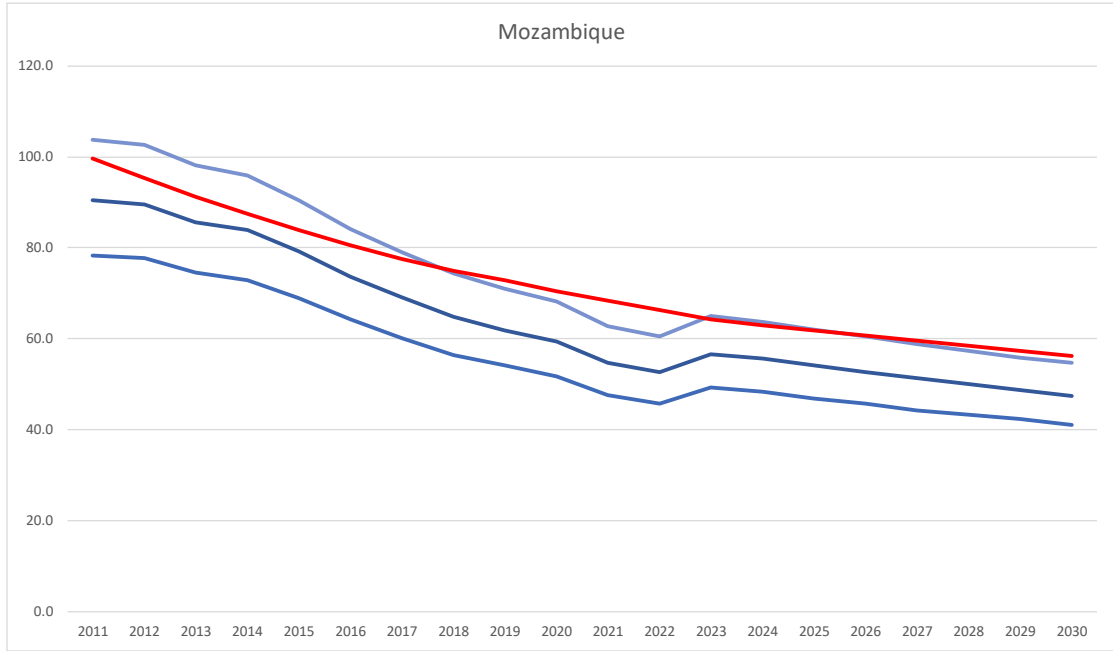

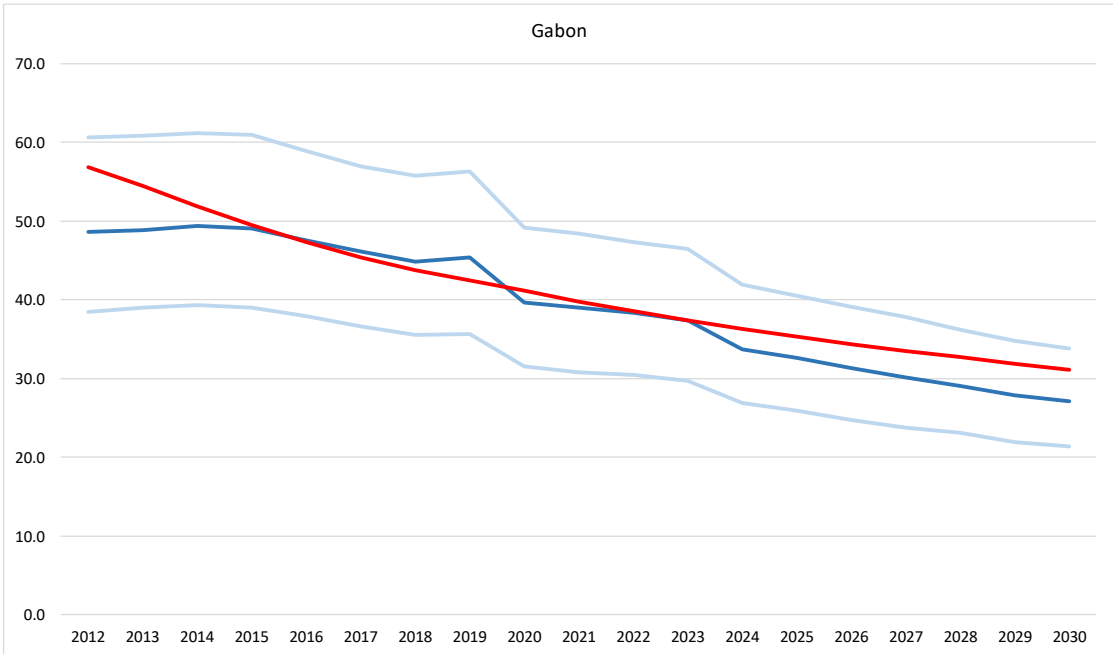

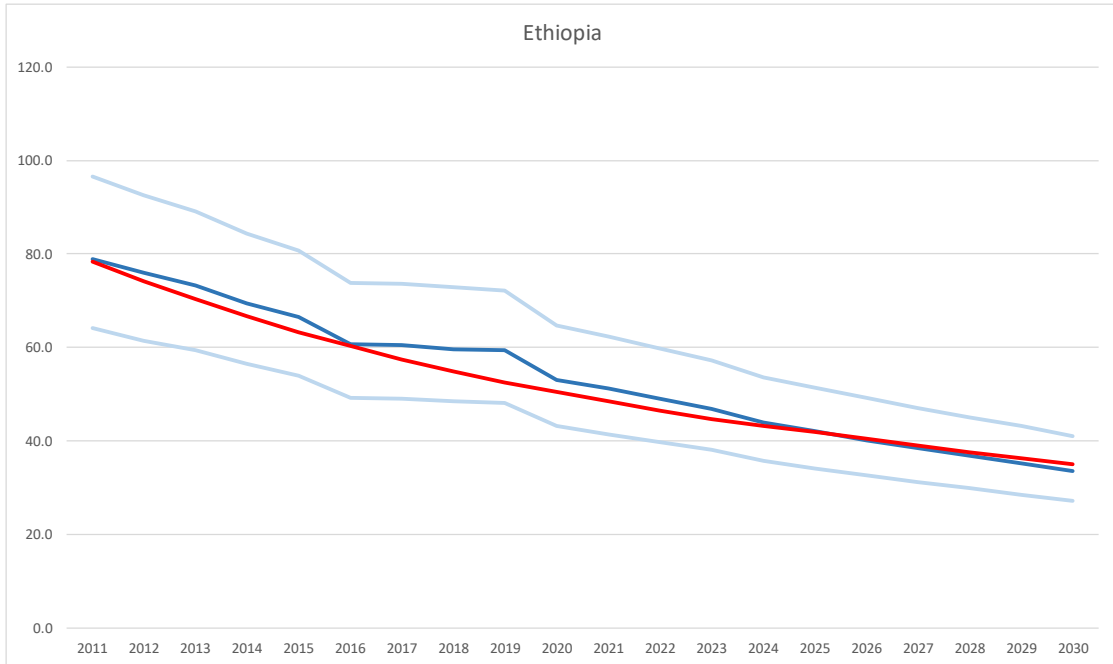

eFigure 3. Validation Data and Graphs, Including U5MR`s (95%UI) Resulted From the Modelling and IGME U5MR's, Period 2011/12 to 2030

eAppendix 5.5. Results

Cohort descriptive statistics

Country	Mozambique				Gabon				Ethiopia			
**Variable**	**2011**	**2020**	**2030**	**2040**	**2012**	**2020**	**2030**	**2040**	**2011**	**2020**	**2030**	**2040**
**Family planning (proportion)**	0.237	0.237	0.237	0.237	0.195	0.195	0.195	0.195	0.314	0.314	0.314	0.314
**Antenatal care (proportion)**	0.468	0.468	0.468	0.468	0.784	0.784	0.784	0.784	0.304	0.304	0.304	0.304
**Skilled birth attendance (proportion)**	0.656	0.656	0.656	0.656	0.957	0.957	0.957	0.957	0.257	0.257	0.257	0.257
**Postnatal neonatal care (proportion)**	0.234	0.234	0.234	0.234	0.664	0.664	0.664	0.664	0.077	0.077	0.077	0.077

© 2026 Barreix G et al. *JAMA Network Open.*

**No perceived barriers in distance and/or money (proportion)**	0.442	0.442	0.442	0.442	0.251	0.251	0.251	0.251	0.257	0.257	0.257	0.257
**Male (proportion)**	0.482	0.482	0.482	0.482	0.525	0.525	0.525	0.525	0.513	0.513	0.513	0.513
**Child´s age (0) (proportion)**	0.257	0.2	0.2	0.2	0.21	0.2	0.2	0.2	0.22	0.2	0.2	0.2
**Child´s age (1) (proportion)**	0.236	0.2	0.2	0.2	0.219	0.2	0.2	0.2	0.193	0.2	0.2	0.2
**Child´s age (2) (proportion)**	0.254	0.2	0.2	0.2	0.175	0.2	0.2	0.2	0.187	0.2	0.2	0.2
**Child´s age (3) (proportion)**	0.105	0.2	0.2	0.2	0.202	0.2	0.2	0.2	0.192	0.2	0.2	0.2
**Child´s age (4) (proportion)**	0.147	0.2	0.2	0.2	0.193	0.2	0.2	0.2	0.208	0.2	0.2	0.2
**Child is part of a twin (proportion)**	0.026	0.026	0.026	0.026	0.027	0.027	0.027	0.027	0.02	0.02	0.02	0.02
**Birth order (1st)**	0.243	0.243	0.243	0.243	0.252	0.252	0.252	0.252	0.184	0.184	0.184	0.184
**Birth order (2nd or 3rd child)**	0.394	0.394	0.394	0.394	0.443	0.443	0.443	0.443	0.306	0.306	0.306	0.306
**Birth order (4th or above child)**	0.363	0.363	0.363	0.363	0.305	0.305	0.305	0.305	0.511	0.511	0.511	0.511
**Maternal age at first birth (>18) (proportion)**	0.508	0.508	0.508	0.508	0.677	0.677	0.677	0.677	0.602	0.602	0.602	0.602
**Maternal education (secondary or higher) (proportion)**	0.105	0.171	0.274	0.379	0.674	0.778	0.964	1	0.037	0.088	0.163	0.229
**Improved form of sanitation (proportion)**	0.217	0.242	0.279	0.324	0.604	0.722	0.94	1	0.128	0.151	0.206	0.254
**Improved source of drinking water (proportion)**	0.352	0.503	0.754	0.912	0.903	0.938	0.985	1	0.475	0.696	0.932	1
**GDP per capita in PPP**	1276	1466	1957	2347	19450	18681	16365	14405	1493	2517	3005	3715
**Percentage of governmental GDP spending on health**	5.0	7.6	9.1	9.1	2.4	3.4	2.5	2.3	4.5	3.5	3.2	3.2
**Gini coefficient**	49.8	50.2	50.4	50.6	39.8	37.0	33.5	30.0	33.6	30.4	27.6	24.9
**National percentage of stunting**	42.6	37.8	35.8	32.9	16.5	13.1	7.3	1.5	44.4	34.7	23.5	12.3
**Number of children**	434,220	572,196	680,856	748,400	269,606	335,980	351,752	394,483	1,572,416	1,880,656	2,136,237	2,239,372

eTable 26. Descriptive PHC Synthetic Cohort Statistics, Baseline and Defunding Scenarios

**Mozambique**	**BAU**	**Defunding**										
		**2025**	**2026**	**2027**	**2028**	**2029**	**2030**	**2031**	**2032**	**2033**	**2034**	**2035**
PHC-score 0	13.0	59.0	80.9	100.0	100.0	100.0	100.0	100.0	100.0	100.0	100.0	100.0
PHC-score 1	21.8	21.8	19.1	0	0	0	0	0	0	0	0	0
PHC-score 2	28.6	19.1	0	0	0	0	0	0	0	0	0	0
PHC-score 3	23.5	0	0	0	0	0	0	0	0	0	0	0
PHC-score 4	11.2	0	0	0	0	0	0	0	0	0	0	0
PHC-score 5	1.9	0	0	0	0	0	0	0	0	0	0	0

© 2026 Barreix G et al. *JAMA Network Open.*

Gabon	BAU	Defunding										
		2025	2026	2027	2028	2029	2030	2031	2032	2033	2034	2035
PHC-score 0	1.2	8.9	16.3	42	82.8	100	100	100	100	100	100	100
PHC-score 1	7.4	7.4	25.7	40.8	17.2	0	0	0	0	0	0	0
PHC-score 2	25.7	25.7	40.8	17.2	0	0	0	0	0	0	0	0
PHC-score 3	40.8	40.8	17.2	0	0	0	0	0	0	0	0	0
PHC-score 4	20.8	17.2	0	0	0	0	0	0	0	0	0	0
PHC-score 5	4.2	0	0	0	0	0	0	0	0	0	0	0
**Ethiopia**	**BAU**	**Defunding**										
		**2025**	**2026**	**2027**	**2028**	**2029**	**2030**	**2031**	**2032**	**2033**	**2034**	**2035**
PHC-score 0	36	56.5	86.5	100	100	100	100	100	100	100	100	100
PHC-score 1	30	30	13.5	0	0	0	0	0	0	0	0	0
PHC-score 2	17.4	13.5	0	0	0	0	0	0	0	0	0	0
PHC-score 3	11	0	0	0	0	0	0	0	0	0	0	0
PHC-score 4	4.8	0	0	0	0	0	0	0	0	0	0	0
PHC-score 5	0.8	0	0	0	0	0	0	0	0	0	0	0

eTable 27. Descriptive Statistics of the PHC-Index by Scenario (Proportion of Under-Five Children in the PHC-Index Category)

BAU= Baseline scenario (this is constant through across the period); Figures are percentages.

Averted Deaths by year and country

	Mozambique			Gabon			Ethiopia		
Year	AD deaths	AD lower	AD upper	AD	AD lower	AD upper	AD	AD lower	AD upper
2025	10171	8800	11751	104	76	137	10657	8530	13121
2026	12588	10740	14681	267	192	344	19444	15610	23963
2027	15652	13390	18070	403	307	512	23444	18919	28780
2028	15485	13180	17940	571	445	717	22554	18260	27513
2029	15195	13070	17610	638	507	792	21701	17800	26840
2030	15006	12860	17310	625	490	779	20723	16667	25240
2031	14824	12639	17080	608	477	769	20001	15990	24701
2032	14652	12580	16860	593	463	748	19280	15419	23890
2033	14390	12270	16642	579	449	731	18572	14919	22750
2034	14175	12280	16441	566	444	707	17914	14320	22020
2035	13943	11860	16230	558	431	702	17227	13800	21340

eTable 28. Averted Deaths (95%UI) (AD) in Baseline vs Defunding Scenario, by Year and Country

eAppendix 5.6. Triangulation Analysis

Population Preventable Fraction (PPF) - Based Estimation of Deaths Averted and Lost Deaths

The PPF is an epidemiological metric that estimates the share of negative outcomes that have been avoided in a population because of a particular exposure. It measures the impact of an intervention at the population level by estimating how much the occurrence of an outcome has

decreased compared to what would have been anticipated if the intervention had not been implemented.

To estimate the potential number of deaths averted between 2025 and 2035, attributable to PHC effective coverage, we used the Population Preventable Fraction (PPF) methods.

We analysed the scenarios in which we compared the *Baseline scenario* vs *Defunding scenario,* as in the main microsimulations based main forecasting analyses. In these scenarios we compare a scenario in which all children stay in current PHC coverage levels vs all children transition to the lowest PHC-index level (PHC-index=0), as defined for the main analyses.

Deaths observed are taken from World Population Prospects (medium variant estimations).

Proportions of children in each PHC-index category across years were taken from the synthetic cohort created, which was based on the last DHS surveys of the countries.

The estimation was done as following:

Let p_i be the proportion of the population in PHC category *i* (0–5), and RR_i the rate ratio for category *i*, with $RR_0 = 1$ (reference category).
The **weighted mean RR** under any distribution is:

$$R\bar{R} = \sum_{i=0}^{5} p_i \cdot RR_i$$

Then Averted deaths/Lives Lost = $D_{obs} \cdot \frac{(\sum RR_b - \sum RR_a)}{\sum RR_{bau}}$

Where Dobs are the observed deaths in BAU, A is the better (lower RR) scenario and B is the worse one, and the denominator is BAU weighted mean RR. Thus, A corresponds to BAU scenario weighted mean RR and b to the Defunding scenario one.

The results are:

Country	Year	Preventable Deaths
**Mozambique**	**2025-2035**	189,204
**Gabon**	**2025-2035**	6,527
**Ethiopia**	**2025-2035**	223,232

eTable 29. Preventable Fraction of the Population Analysis

© 2026 Barreix G et al. *JAMA Network Open.*

Generalizability of the Results

This study examined three diverse countries in SSA—Mozambique, Ethiopia, and Gabon—providing a lens through which to analyse the region's diverse dynamics. These nations reflect geographical and subregional variation across SSA, encompassing Southern Africa, East Africa, and Central Africa. Additionally, they exhibit heterogeneity in population scales and distinct stages in their demographic transition. While Mozambique and Ethiopia are characterized as low-income, agriculture-based economies, Gabon stands as an upper-middle-income nation with a resource-rich, oil-dependent economy. The countries also exhibit distinct U5M causes epidemiological profiles within the SSA region. In Gabon, malaria remains a predominant health concern, whereas in Ethiopia its burden is comparatively lower, with Mozambique aligning closer to the regional average. Conversely, Ethiopia experiences a greater prevalence of neonatal disorders. Gabon, likely due to improved water, sanitation, and hygiene infrastructure, demonstrates lower susceptibility to diarrheal diseases, while Mozambique bears a significantly higher HIV burden. These variations offer a valuable comparative framework for assessing health outcome heterogeneity across the region.[1, 52, 53]

The heterogeneity among Gabon, Mozambique, and Ethiopia is further evident with respect to their PHC systems. In Gabon, the delivery of PHC services is predominantly centred around hospital infrastructure. In contrast, Mozambique has implemented a more geographically decentralized model, with a network of health centres and posts that is being progressively strengthened through the large-scale integration of community health workers. Ethiopia, in turn, has undertaken a paradigm-shifting approach within the region, significantly expanding its PHC system over the past decade. This expansion has involved substantial investments in PHC hospitals, centres, and posts, in an integrated manner alongside a marked increase in the health workforce through the training of community health workers and nurses.[14, 21, 23, 54-57]

Lastly, these countries represent distinct realities in terms of DAH relative to their total health expenditure. This distinction is particularly important given the ongoing disruptions in DAH funding.[1]

The diversity observed across Mozambique, Ethiopia, and Gabon offers a valuable basis for interpreting the broader landscape of PHC association with child mortality in the SSA region. Their contrasts in geography, epidemiological profiles, PHC system organisation, and levels of development assistance for health provide a wide analytical window that strengthens the plausibility of extending our insights to the wider region. At the same time, this heterogeneity does not equate to statistical representativeness, and caution is warranted when extrapolating findings to all SSA countries. The analysis design supports a more nuanced generalisability understanding towards the region, rather than an unequivocal region-wide claim.

eReferences.

1. Institute for Health Metrics and Evaluation. Institute for Health Metrics and Evaluation. https://www.healthdata.org. Accessed May 20, 2026.
2. Hone T, Macinko J, Millett C. Revisiting Alma-Ata: what is the role of primary health care in achieving the Sustainable Development Goals? Lancet. 2018;392(10156):1461-1472.
3. World Health Organization. Implementing the Primary Health Care Approach: A Primer. World Health Organization; 2024.
4. Bhutta ZA, Ali S, Cousens S, et al. Alma-Ata: rebirth and revision 6—interventions to address maternal, newborn, and child survival: what difference can integrated primary health care strategies make? Lancet. 2008;372(9642):972-989.
5. World Health Organization, United Nations Children's Fund. Primary Health Care Measurement Framework and Indicators: Monitoring Health Systems Through a Primary Health Care Lens. World Health Organization; 2021.
6. Ahinkorah BO, Ameyaw EK, Seidu AA, Odusina EK, Keetile M, Yaya S. Examining barriers to healthcare access and utilization of antenatal care services: evidence from demographic health surveys in sub-Saharan Africa. BMC Health Serv Res. 2021;21:1-16.
7. Tessema ZT, Worku MG, Tesema GA, et al. Determinants of accessing healthcare in sub-Saharan Africa: a mixed-effect analysis of recent Demographic and Health Surveys from 36 countries. BMJ Open. 2022;12(1):e054397.
8. Negash HK, Endale HT, Aragie H, et al. Barriers to healthcare access among female youths in Mozambique: a mixed-effects and spatial analysis using DHS 2022/23 data. BMC Public Health. 2025;25:498.
9. Adugna MB, Nabbouh F, Shehata S, Ghahari S. Barriers and facilitators to healthcare access for children with disabilities in low and middle income sub-Saharan African countries: a scoping review. BMC Health Serv Res. 2020;20:1-11.
10. Ministry of Health of Ethiopia. Health and Health Related Indicators 2016 EFY (2023/2024 GC): The 26th Annual Review Meeting of the Health Sector. Ministry of Health of Ethiopia; 2024.
11. Hossain S, Christopoulos C. Ethiopia's Health Extension Program.
12. Ethiopian Public Health Institute. Health Sector Transformation Plan-I (HSTP-I): 2015/16-2019/20. Ethiopian Public Health Institute; 2022.
13. Hierink F, Oladeji O, Robins A, Muñiz MF, Ayalew Y, Ray N. A geospatial analysis of accessibility and availability to implement the primary healthcare roadmap in Ethiopia. Commun Med (Lond). 2023;3(1):140.
14. Ministry of Health of Ethiopia. Health Sector Transformation Plan II (HSTP II). Ministry of Health of Ethiopia; 2021.

© 2026 Barreix G et al. *JAMA Network Open.*

15. Mouelet AM, El-Idrissi MDZE, Robyn PJ. Gabon Indigents Scheme: A Social Health Insurance Program for the Poor. World Bank; 2018.

16. USAID. Gabon: health. USAID. https://www.usaid.gov. Accessed May 20, 2026.

17. Africa Health Business. Gabon's health sector. Africa Health Business.

18. World Health Organization. The Global Health Observatory. WHO. https://www.who.int/data/gho. Accessed May 20, 2026.

19. World Bank. World Bank Open Data. World Bank. https://data.worldbank.org. Accessed May 20, 2026.

20. Aboubacar I, Essono M, Barroy H, Mailfert M. Health Financing and Budgeting Reforms in Gabon: Process and Challenges on the Road to Universal Health Coverage. WHO; 2020.

21. Borde E, Martínez-Herrera E, Navarro MGZ, et al. Financiamento do Sistema de Saúde de Moçambique. [s.n.]; 2022.

22. World Health Organization. Mozambique. WHO. https://www.who.int/mozambique. Accessed May 20, 2026.

23. Ministry of Health of Mozambique. Health Sector Strategic Plan 2014-2019. Ministry of Health of Mozambique; 2014.

24. World Bank. Mozambique's health system expands coverage with more frontline community workers. World Bank. https://www.worldbank.org. Accessed May 20, 2026.

25. República de Moçambique Ministério da Saúde, Direcção de Planificação e Cooperação. Plano Estratégico do Sector da Saúde PESS 2014-2019 (Extensão 2020-2024). MISAU; 2022.

26. Bitton A, Ratcliffe HL, Veillard JH, et al. Primary health care as a foundation for strengthening health systems in low- and middle-income countries. J Gen Intern Med. 2017;32:566-571.

27. Mejía-Guevara I, Zuo W, Bendavid E, Li N, Tuljapurkar S. Age distribution, trends, and forecasts of under-5 mortality in 31 sub-Saharan African countries: a modeling study. PLoS Med. 2019;16(3):e1002757.

28. Global Conference on Primary Health Care. Declaration of Astana. WHO/UNICEF; 2018.

29. Balaj M, York HW, Sripada K, et al. Parental education and inequalities in child mortality: a global systematic review and meta-analysis. Lancet. 2021;398(10300):608-620.

30. Srivastava S, Upadhyay SK, Chauhan S, Alagarajan M. Preceding child survival status and its effect on infant and child mortality in India: evidence from National Family Health Survey 2015-16. BMC Public Health. 2021;21(1):1577.

© 2026 Barreix G et al. *JAMA Network Open.*

31. Kaldewei C. Determinants of Infant and Under-Five Mortality—the Case of Jordan. Technical Note. United Nations Department of Economic and Social Affairs; 2010.
32. USAID. The DHS Program. USAID. https://dhsprogram.com. Accessed May 20, 2026.
33. Talbot D, Mésidor M, Chiu Y, Simard M, Sirois C. An alternative perspective on the robust Poisson method for estimating risk or prevalence ratios. Epidemiology. 2023;34(1):1-7.
34. Höfler M. The Bradford Hill considerations on causality: a counterfactual perspective. Emerg Themes Epidemiol. 2005;2:1-9.
35. Hone T, Gonçalves J, Seferidi P, et al. Progress towards universal health coverage and inequalities in infant mortality: an analysis of 4.1 million births from 60 low-income and middle-income countries between 2000 and 2019. Lancet Glob Health. 2024;12(5):e744-e755.
36. Moncayo AL, Cavalcanti DM, Ordoñez JA, et al. Can primary health care mitigate the effects of economic crises on child health in Latin America? An integrated multicountry evaluation and forecasting analysis. Lancet Glob Health. 2024;12(6):e938-e946.
37. Rasella D, Aquino R, Santos CAT, Paes-Sousa R, Barreto ML. Effect of a conditional cash transfer programme on childhood mortality: a nationwide analysis of Brazilian municipalities. Lancet. 2013;382(9886):57-64.
38. Macinko J, Guanais FC, de Souza MDFM. Evaluation of the impact of the Family Health Program on infant mortality in Brazil, 1990-2002. J Epidemiol Community Health. 2006;60(1):13-19.
39. Islam MA, Tabassum T. Does antenatal and post-natal program reduce infant mortality? A meta-analytical review on 24 developing countries based on demographic and health survey data. Sex Reprod Healthc. 2021;28:100616.
40. Oduse S, Zewotir T, North D. The impact of antenatal care on under-five mortality in Ethiopia: a difference-in-differences analysis. BMC Pregnancy Childbirth. 2021;21:1-9.
41. Binka FN, Nazzar A, Phillips JF. The Navrongo community health and family planning project. Stud Fam Plann. 1995;26(3):121-139.
42. Adedini SA, Odimegwu C, Imasiku EN, Ononokpono DN. Unmet need for family planning: implication for under-five mortality in Nigeria. J Health Popul Nutr. 2015;33(1):187.
43. Amouzou A, Ziqi M, Carvajal-Aguirre L, Quinley J. Skilled attendant at birth and newborn survival in sub-Saharan Africa. J Glob Health. 2017;7(2):020504.

© 2026 Barreix G et al. *JAMA Network Open.*

44. Tekelab T, Chojenta C, Smith R, Loxton D. The impact of antenatal care on neonatal mortality in sub-Saharan Africa: a systematic review and meta-analysis. PLoS One. 2019;14(9):e0222566.

45. World Health Organization. Global Health Expenditure Database. WHO. https://apps.who.int/nha/database. Accessed May 20, 2026.

46. Debusmann B Jr. More than 80% of USAID programmes "officially ending." BBC News. March 10, 2025. https://www.bbc.com. Accessed May 20, 2026.

47. Osendarp S, Ruel M, Udomkesmalee E, Tessema M, Haddad L. The full lethal impact of massive cuts to international food aid. Nature. 2025;640(8057):35-37.

48. Cavalcanti DM, de Oliveira Ferreira de Sales L, da Silva AF, et al. Evaluating the impact of two decades of USAID interventions and projecting the effects of defunding on mortality up to 2030: a retrospective impact evaluation and forecasting analysis. Lancet. 2025 Jul 1. doi:10.1016/S0140-6736(25)01186-9.

49. Caro JJ, Briggs AH, Siebert U, Kuntz KM. Modeling good research practices—overview: a report of the ISPOR-SMDM Modeling Good Research Practices Task Force–1. Med Decis Making. 2012;32(5):667-677.

50. United Nations Department of Economic and Social Affairs Population Division. World Population Prospects 2024. United Nations; 2024.

51. United Nations Inter-agency Group for Child Mortality Estimation. Levels and Trends in Child Mortality: Report 2024—Estimates Developed by the United Nations Inter-agency Group for Child Mortality Estimation. UNICEF; 2025.

52. World Bank. Mozambique overview. World Bank. https://www.worldbank.org/en/country/mozambique/overview. Accessed March 18, 2025.

53. FERDI. Comparing Sub-Saharan Africa and upper middle income countries. FERDI Competitiveness Observatory. https://competitivite.ferdi.fr/en/countries/compare/sub-saharan-africa/upper-middle-income-countries. Accessed March 21, 2025.

54. World Health Organization. PRIMARY HEALTH CARE SYSTEMS (PRIMASYS) Case study from Ethiopia WHO; 2017. https://iris.who.int/bitstream/handle/10665/341083/WHO-HIS-HSR-17.31-eng.pdf. Accessed May 20, 2026.

55. London School of Hygiene and Tropical Medicine. SCOPING REPORT Financing Primary Health Care in Ethiopia LSHTM. https://www.lshtm.ac.uk/media/59786. Accessed May 20, 2026.

56. World Health Organization. HEALTH FINANCING AND BUDGETING REFORMS IN GABON: PROGRESS AND CHALLENGES ON THE ROAD TO UNIVERSAL HEALTH COVERAGE WHO; 2020.

© 2026 Barreix G et al. *JAMA Network Open.*

https://iris.who.int/bitstream/handle/10665/333604/9789240006881-eng.pdf. Accessed May 20, 2026.

57. MISAU Plano Nacional De Desenvolvimento De Recursos Humanos Para A Saúde 2016-2025). 2016.

© 2026 Barreix G et al. *JAMA Network Open.*
