# Supplementary material for: Primary Health Care Coverage and Child Mortality in Sub-Saharan Africa
Source: JAMA Netw Open. 2026 Aug 7;9(8):e2627655. doi: 10.1001/jamanetworkopen.2026.27655 (PMC13451977; doi:10.1001/jamanetworkopen.2026.27655)
Supplement: Supplementary file 2 [file jamanetwopen-e2627655-s002.pdf]

Data Sharing Statement

Barreix. Primary Health Care Coverage and Child Mortality in Sub-Saharan Africa. *JAMA Netw Open*. Published August 07, 2026. doi:10.1001/jamanetworkopen.2026.27655

Data
Data available: Yes
Data types: Deidentified participant data
How to access data: The data used for the study is available in https://dhsprogram.com/
When available: With publication

Supporting Documents
Document types: None

Additional Information
Who can access the data: The data used for this study is all publicly available. In case of any particular requirement, it will be available upon request.
Types of analyses: Upon request
Mechanisms of data availability: Upon agreement
Any additional restrictions: Nothing to declare at this stage
